# Prolonged DEHP exposure enhances the stemness and metastatic potential of TNBC cells in an MSI2-dependent manner

**DOI:** 10.7150/ijbs.101598

**Published:** 2025-02-03

**Authors:** Mahendra Jadhao, Sheng-Kai Hsu, Dhanashri Deshmukh, Pei-Feng Liu, Shih-Feng Weng, Yih-Fung Chen, Chia-Yang Li, Chia-Yih Wang, Eing-Mei Tsai, Li-Fang Wang, Chien-Chih Chiu

**Affiliations:** 1 Department of Medicinal and Applied Chemistry, Kaohsiung Medical University, Kaohsiung 807, Taiwan.; 2 Department of Cancer Biology, University of Cincinnati College of Medicine, Cincinnati 45220, OH, USA.; 3 Department of Biotechnology, Kaohsiung Medical University, Kaohsiung 807, Taiwan.; 4 Department of Biomedical Science and Environmental Biology, Kaohsiung Medical University, Kaohsiung 807, Taiwan.; 5 Department of Healthcare Administration and Medical Informatics, Kaohsiung Medical University, Kaohsiung 807, Taiwan.; 6 Graduate Institute of Natural Products, Kaohsiung Medical University, Kaohsiung 807, Taiwan.; 7 Graduate Institute of Medicine, College of Medicine, Kaohsiung Medical University, Kaohsiung 80708, Taiwan.; 8 Department of Cell Biology and Anatomy, College of Medicine, National Cheng Kung University, Tainan 704, Taiwan.; 9 Department of Obstetrics and Gynecology, Kaohsiung Medical University Hospital, Kaohsiung 807, Taiwan.; 10 Department of Biological Sciences, National Sun Yat-Sen University, Kaohsiung, 804, Taiwan.; 11 Center for Cancer Research, Kaohsiung Medical University Hospital, Kaohsiung Medical University, Kaohsiung 807, Taiwan.; 12 Department of Medical Research, Kaohsiung Medical University Hospital, Kaohsiung 807, Taiwan.

**Keywords:** Triple-negative breast cancer (TNBC), Di-2-ethylhexyl phthalate (DEHP), Musashi RNA binding protein 2 (MSI2), Metastasis, Stemness, miR-155-5p

## Abstract

Di(2-ethylhexyl) phthalate (DEHP) is a commonly used plasticizer, and human exposure to phthalates is a major health concern. DEHP, which is widely recognized as an endocrine disruptor, is associated with an increased risk of several diseases, including breast cancer. Triple-negative breast cancer (TNBC) is an aggressive subtype of breast cancer, and metastasis is the leading cause of TNBC-related mortality. However, the correlation between DEHP exposure and TNBC metastasis remains elusive. In the present study, we found that prolonged DEHP treatment enhanced the migration and invasion of TNBC cells both *in vitro* and *in vivo*. Mechanistically, DEHP exposure induced Musashi RNA binding protein 2 (MSI2) overexpression, which subsequently activated the PI3K/Akt/NF-κB/MMP-9 axis to augment metastatic potential. MSI2 also promoted stemness. Interestingly, we identified a novel function of MSI2 in regulating the expression, distribution, and polarization of vimentin that is independent of its conventional RNA binding and translation regulation. Genetic knockdown of MSI2 potently abolished DEHP-mediated TNBC progression. Moreover, MSI2 depletion inhibited lung metastasis in metastatic mouse models but did not affect proliferation or tumor size. Intriguingly, miR-155-5p downregulation was observed after DEHP exposure, while mimic miR-155-5p treatment inhibited DEHP-induced TNBC migration, accompanied by reduced expression of MSI2 and vimentin. These findings suggested an inverse relationship between miR-155-5p levels and MSI2 expression. Taken together, MSI2 might serve as a potential therapeutic target and function as a prognostic biomarker for TNBC patients.

## Introduction

Phthalates are commonly added to improve the flexibility and durability of plastic products. Among phthalates, di(2-ethylhexyl) phthalate (DEHP) is ubiquitous in medical devices, food packaging, personal care products, and the cosmetic industry and accounts for 84% of all phthalate production 1. DEHP, which is widely recognized as an endocrine disruptor, is detrimental to multiple organs (e.g., neurotoxicity, reprotoxicity, and nephrotoxicity) 2-4. Furthermore, prolonged DEHP exposure is closely related to increased risks of several disorders, including infertility, neurodegenerative diseases, and malignancy 5-7. Indeed, an increasing number of studies have revealed that DEHP exposure potently induces tumorigenesis 8-10. In non-small cell lung carcinoma (NSCLC), DEHP impaired the antitumor activity of camptothecin by upregulating the Akt/NF-κB axis and alleviating DNA damage 9. Even at low concentrations (10^-8^~10^-6^ M), DEHP can also increase the proliferation of MCF-7 cells by activating PI3K/Akt signaling 11. Overall, human DEHP exposure is a major public health concern.

Breast cancer (BC) is not only the most prevalent but also the second leading cause of cancer-related mortality in female populations following lung and bronchus cancer 12. Triple-negative breast cancer (TNBC) is an aggressive subtype of BC with a poor prognosis due to the lack of specific therapeutic targets, including human epidermal growth factor receptor 2 (HER-2) and hormone receptors, which renders treatment more challenging 13. According to an observational study, the five-year overall survival (OS) rate of patients with TNBC is 81.28%, but that of patients with metastatic TNBC is merely 10.81%; this highlights that metastasis is the main cause of TNBC-related death 14. Our previous studies revealed that prolonged DEHP exposure induced multidrug resistance and angiogenesis in TNBC through the upregulation of ATP-binding cassette transporters and the endoglin-dependent pathway, respectively 15, 16. Nevertheless, the relationship between DEHP exposure and the metastatic capacity of TNBC remains elusive.

Musashi RNA binding protein 2 (MSI2) was first identified in *Drosophila melanogaster* and is a member of the RNA-binding protein-encoding gene family. Physiologically, MSI2 is involved in self-renewal and is ubiquitously expressed in the progenitor cells of hematopoiesis and the nervous system 17. Increasing evidence suggests that MSI2 functions as an oncogene to support the metastatic behavior of several malignancies. Chen *et al.* reported that MSI2 overexpression was closely related to liver metastasis in patients with colorectal cancer 18. MSI2 enhances the migration and invasion of bladder cancer cells by activating the JAK2/STAT3 axis 19. Additionally, MSI2 induces epithelial‒mesenchymal transition (EMT) in esophageal squamous cell carcinoma by activating Wnt/β-catenin signaling 20. These studies indicate that MSI2 plays a critical role in facilitating cancer metastasis by regulating diverse mechanisms. Nevertheless, the involvement of MSI2 in TNBC metastasis has been relatively less investigated.

In the present study, we explored the effects of long-term DEHP exposure on TNBC progression. Our results demonstrated that DEHP treatment promoted the stemness, migration, invasion, and distant metastasis of TNBC cells. Mechanistically, DEHP-mediated MSI2 overexpression induced metastasis via the PI3K/Akt/NF-κB/MMP-9 axis. Intriguingly, a novel function of MSI2 (an RNA-binding protein) in regulating the expression, distribution, and polarization of vimentin was identified. Overall, our current study provides novel insight into the relationship between environmental contamination and TNBC progression, which highlights that MSI2 might serve as a potential therapeutic target and prognostic biomarker specifically for patients with metastatic TNBC.

## Materials and Methods

### Cell cultures

The Hs578T, MCF7, and MDA-MB-231 human breast cancer cell lines were obtained from the American Type Culture Collection (ATCC, VA, USA) and cultured in DMEM (Gibco, NY, USA) supplemented with 10% fetal bovine serum (FBS), 0.03% glutamine, 1 mM sodium pyruvate, and the antibiotics penicillin/streptomycin (100 μg/mL). Human mammary epithelial cells (HMECs) (#CC-2251, Lonza, Basel, Switzerland) were cultured in MEBM™ Mammary Epithelial Cell Growth (MEGM) basal medium (CC-4136) with the MEGM SingleQuots^TM^ Kit (#CC-3150, Lonza). 293T cells (ATCC) were cultured in low-antibiotic (0.1%) DMEM/F12 supplemented with 10% FBS. All the cells were maintained in an incubator containing 5% CO_2_ at 37 °C.

### Reagents and antibodies

DEHP (#36735, Sigma-Aldrich, St. Louis, Missouri, USA) was diluted with DMSO (Sigma). Thiazolyl blue tetrazolium bromide (MTT) reagent (#M5655-500MG, Sigma-Aldrich) was stored at -20 °C until use. Doxorubicin (#D1515, Dox, Sigma-Aldrich) was dissolved in DMSO (Sigma-Aldrich) and stored at -20 °C until use. Primary antibodies targeting the following proteins were used: MSI2 (37 kDa, Proteintech, 10770-1-AP), β-catenin (92 kDa, Proteintech, 51067-2-AP), vimentin (54 kDa, Gentex, GTX110619), α-SMA (46 kDa, Abcam, ab32575), SNAI 1 (28 kDa, CusAb, CSB-PA021867LA01HU), CD133 (115 kDa, Proteintech, 18470-1-AP), c-Myc (69 kDa, Abcam, ab32072), SOX2 (35 kDa, CusAb, PA16539AORb), HLA class I ABC (41 kDa, Proteintech, 66013-1-Ig), β-actin (45 kDa, Santa Cruz, SC-47778), phospho-PI3K p85 (Tyr458) (85 kDa, Cell Signaling, 4228t), PI3 kinase p85 (85 kDa, Cell Signaling, 4257s), phospho-AKT (Ser473) (60 kDa, Cell Signaling, 4060s), AKT (60 kDa, Cell Signaling, 4691s), Ikkα (85 kDa, Cell Signaling, 2682s), Ikkβ (87 kDa, Cell Signaling #2684), and Ikkε (80 kDa, Cell Signaling, 2690s), phospho-Ikkα/β (85 kDa, Santa Cruz, SC-23470-R), NFκB p65 (65 kDa, Cell Signaling, 8242s), Lamin A/C (74/63 kDa, Cell Signaling, 4777s), GAPDH (35.5 kDa, EMD Millipore, MAB374), vimentin (54 kDa, BD Bioscience, BD550513), Ki67 (395/347 kDa, Santa Cruz, SC23900), TNFα (26 kDa, Gentex, GTX115020) and PCNA (36 kDa, Santa Cruz, SC56). The secondary antibodies used were goat anti-mouse IgG (Alexa Fluor 488, #A11001) and goat anti-rabbit IgG (Alexa Fluor 594, #A11012), which were purchased from Invitrogen.

### Establishment of cell lines with long-term DEHP exposure

MCF7, Hs578T, MDA-MB-231 and normal HMECs were exposed to a low concentration of DEHP (100 nM) for three months as described previously 15. Subsequently, DEHP was withdrawn, and the cells were challenged with Dox (10 nM) for 72 h. Following this treatment, the cells were subjected to a colony formation assay. Colonies grown after Dox treatment were selected and expanded separately (2 drug-resistant clones, #1 and #2, which were generated from MDA-MB-231 cells, were used throughout the study to maintain the experimental replicates).

### Wound healing assay

The effects of prolonged DEHP treatment on the mobility of normal HMECs and tumorigenic Hs578T, MCF7, and MDA-MB-231 cells were assessed via a wound healing assay. In brief, all untreated and DEHP-exposed cells were cultured on a 12-well plate until a confluent monolayer formed, and a 100-µl pipette tip was used to form a straight wound, which was subsequently washed once with medium. Images of the scratch were captured at 0 h and after 12, 20, and 24 h for HMECs, MCF7 cells, Hs578T cells, and MDA-MB231 cells (clones #1 and #2), respectively. These images were analyzed via the software programs TScratch (v1.0, CSElab, ETH Zurich) and SigmaPlot (v12.3, Systat Software Inc., Germany).

### Cell invasion assay

A total of 1x10^5^ untreated and DEHP-exposed MCF7, Hs578T, MDA-MB-231, and normal HMEC cells were cultured with serum-free medium in the transwell inserts of 24-well Transwell inserts (Greiner Bio-One, 662638, Switzerland) with 8-µm-pore membranes coated with Cultrex UltraMatrix Reduced Growth Factor (RGF) Basement Membrane Extract matrix (R&D, #BME001-05). The inserts were placed in 24-well plates containing complete medium, and HMECs, MCF7 cells, Hs578T cells, and MDA-MB231 cells (clones #1 and #2) were allowed to invade for 12, 20, and 24 h, respectively. The migrated cells were fixed with paraformaldehyde and stained with Giemsa. The cells were counted in at least five microscopic fields per transwell insert. SigmaPlot was used to calculate the cell density.

### Cell proliferation

An MTT assay was performed as described previously 21. Briefly, 1.5x10^2^ and 2.5x10^2^ MDA-MB-231 parental cells and DEHP-exposed clones were seeded in 96-well plates and incubated for 24 h and 48 h, respectively. Afterwards, the cells were washed with PBS, treated with 100 μl of medium containing MTT (0.5 mg/ml), and incubated at 37 °C in the dark for 3 h. The media were replaced with DMSO (100 µl/well), and the plates were incubated on a shaker at 37 °C in the dark. The absorbance of each well was read via a microplate reader (BioTek-800^TS^, BioTek Inc., USA). SigmaPlot was used for data analysis and quantification.

### Zebrafish xenograft assay

Transgenic Tg (fli1: EGFP) zebrafish were maintained and bred at the Core Zebrafish Facility at Kaohsiung Medical University. Tg (fli1: EGFP) zebrafish embryos at 48 hpf (n=50) were injected with 4 x10^6^ DiI (Thermo Fisher, V22885)-stained untreated and DEHP-exposed Hs578T and MDA-MB-231 (clone #1) cells into the embryo sac. The embryos were imaged under a fluorescence microscope (Leica, MZ10F, Buckinghamshire, UK) 24 h postinjection (24 hpi). The number of embryos showing cell migration to subintestinal vessels (SIVs) was recorded and analyzed. ImageJ software (ImageJ, NIH, Maryland, USA) was used to analyze the fluorescence images to evaluate the migration efficiency of the injected cells. The above *in vivo* experiments were approved by the Institutional Animal Care and Use Committee (IACUC), Kaohsiung Medical University, Kaohsiung, Taiwan (#108182). The data were quantified via SigmaPlot.

### Library preparation and transcriptome sequencing

TRIzol^TM^ reagent (Thermo Fisher, 10296010) was used for RNA extraction from parental MDA-MB-231 cells, DEHP-exposed clones, and Dox-resistant (DoxR) cells. Purified RNA samples were commissioned to Biotools Co., Ltd. (Taipei, Taiwan) for library preparation and sequencing 15. Briefly, RNA library preparation was performed, and gene mapping and analysis of differentially expressed genes (DEGs) were conducted via TopHat (v2.0.12). Gene Ontology (GO) enrichment was analyzed via topGO and GoSeq (Release 2.12), and Kyoto Encyclopedia of Genes and Genomes (KEGG) analysis was performed to identify enriched biological pathways via KOBAS (v2.0), which was validated via ingenuity pathway analysis (IPA).

### The Cancer Genome Atlas (TCGA) analysis

RNA-sequencing data of the transcriptome of breast cancer patients were acquired and analyzed from The Cancer Genome Atlas (TCGA) database (https://cancergenome.nih.gov). Statistical analysis of the TCGA data was analyzed using the SPSS software (v20.0, IBM-SPSS Inc., Chicago, IL, USA). In the TCGA cohort, patients were dichotomized on the basis of low or high MSI2 expression, with the cutoff value on the basis of the ROC curve. Univariate analyses were used for crude and adjusted hazard ratios (CHRs and AHRs). A value of *p* ≤ 0.05 was considered significant.

### Immunoblotting

Immunoblotting was performed to evaluate changes in protein levels as described previously 22. Briefly, a Pierce BCA protein estimation kit (Thermo Fisher, #23225) was used to quantify proteins, which were then separated by SDS‒polyacrylamide gel electrophoresis (SDS‒PAGE) and transferred to PVDF membranes, which were incubated with primary antibodies before they were washed and incubated with secondary antibodies conjugated with HRP. The blots were incubated with ECL^TM^ reagent, and chemiluminescence signals corresponding to the protein bands were acquired using an Amersham Imager 680 (GE Healthcare Bio-Sciences, Uppsala, Sweden).

### Soft agar colony formation assay

To evaluate the effects of prolonged DEHP exposure on breast cancer cell growth, stem cell-like anchorage-independent (AIG) growth was assessed using a soft agar colony/spheroid formation assays. Briefly, 1.4% low-melting agarose (IBI Scientific, IB70051, USA) was dissolved in distilled water and autoclaved. Six-well plates were coated with 300 µl of 0.7% agar in 2× DMEM (1:1 ratio of 1.4% agarose to 2× DMEM). The coated plates were incubated in a laminar flow hood until the agarose layer solidified. A total of 2x10^2^ untreated and DEHP-exposed MDA-MB-231 cell clones were suspended in DMEM and 0.7% agarose solution (1:1 ratio), and 300 µl per well was plated on a 0.7% agarose layer and allowed to solidify for 30 min under laminar flow. The plates were incubated for 10-12 days at 37 °C supplemented with complete cell culture medium (200-300 µl) twice a week. After incubation, all the colonies or spheroids were counted. ImageJ software (NIH, Maryland, USA) was used to analyze the colony/spheroid sizes, and Prism 9 software (GraphPad Software, California, USA) was used for quantitative analysis.

### Lentiviral transfection

Lentiviral particles were prepared by a packaging plasmid (pCMV-dR8.91), an envelope plasmid (pMD2. G), and the pLKO.1 vector with the cloned shRNAs of interest. MSI2 shRNA and scrambled shRNA (Table 1) were obtained from the RNAi core facility (Academia Sinica, Taipei, Taiwan). 293T cells (1x105) were seeded into a 6-well plate and incubated overnight. The packaging plasmids and plasmids containing shRNAs were mixed with OptiMEM and Lipofectamine 2000 (Thermo Fisher, #11668019). The transfection mixture was added to the 293T cells and incubated for 18 h. The medium was replaced with DMEM supplemented with 1% BSA, and the lentivirus was harvested at 36 h and 48 h after transfection and concentrated via a Macrosep^®^ 100K centrifugal device (Pall Corporation, MAP100C38). Untreated and DEHP-exposed MDA-MB-231 (clones #1 and #2) cells were infected with shScr- or shMSI2-containing lentivirus and incubated for 24 h. Transfected cells were selected by treatment with puromycin (1-2 µg/ml) for sub- cloning stable MSI2 knockdown cells.

### RNA-IP and transcriptome sequencing

MSI2, an RNA-binding protein, interacts with intracellular RNAs, including mRNAs, to control gene expression. To evaluate the RNAs that interact with MSI2, RNA immunoprecipitation (RNA-IP) was conducted via an RNA-Binding Protein Immunoprecipitation Kit (#17-701, EZ-Magna RIP^TM^, Merck) with an MSI2 antibody as bait to capture MSI2-RNA complexes according to the manufacturer's protocol. Briefly, 5-7 µg of MSI2 antibody (Proteintech, 10770-1-AP) was conjugated to magnetic beads. A total of 2.5x10^7^ untreated MDA-MB-231 parental and DEHP-exposed clones were treated with RIPA lysis buffer. MSI2-antibody-conjugated beads were incubated with cell lysates containing protein complexes along with RIP buffer on a mixer. The beads were treated with proteinase K in SDS buffer to separate the RNA from the protein‒RNA complexes, and the RNA was purified. The upper aqueous phase was mixed with chloroform and centrifuged at 14000 rpm. RNA was precipitated by sequentially mixing with salt solution 1, salt solution 2 and the precipitate enhancer in absolute ethanol. The samples were stored at -80 °C overnight for precipitation and centrifuged the following day to collect RNA pellets, which were subsequently washed with 80% ethanol and redissolved in RNase-free water. The RNA samples were sent to Albie Life, Inc. (Taichung, Taiwan) for RNA sequencing and library preparation, which were performed as described previously 23.

### NFκB immunofluorescence

A total of 2x10^4^ untreated MDA-MB-231 and DEHP-exposed clone #1 cells were seeded on circular cover slips in 24-well plates. Upon reaching confluence, the cells were washed with PBS and PFA-fixed prior to permeabilization with 0.5% Triton X-100 (Sigma-Aldrich, X100). After the cells were blocked with 3% BSA, they were incubated with an NFκB p65 antibody (Cell Signaling, 8242s) at 4 °C overnight, washed and incubated with a fluorescence-conjugated secondary antibody (Invitrogen, A32740) for 2 h at room temperature. The cells were stained with the nuclear staining dye DAPI and mounted on glass slides using Bidi mounting medium (ibidi GmbH, Grafelfing, Germany). Fluorescence images viewed on a fluorescence microscope (Olympus, 1X71) were acquired, and the data were analyzed using ImageJ software and quantified using SigmaPlot.

### NFκB p65 inhibitor (BAY11-7082) treatment

To validate the role of NFκB p65 in MMP9 regulation, cells were treated with the NFκB p65 inhibitor BAY11 7082. Briefly, 5x10^5^ untreated MDA-MB-231 and DEHP-exposed clone #1 cells were seeded in 60-mm Petri dishes and incubated at 37 °C overnight, after which they were washed with 1X PBS and treated with the NFκB p65 inhibitor BAY11--7082 (10 µM) for 24 h. Afterwards, the cells were washed, trypsinized and processed for total and nuclear protein extraction, NFκB p65 immunofluorescence (IF), and wound healing assays.

### Gelatin zymography

Scramble-treated MDA-MB-231 cells, DEHP-exposed clones, MSI2-depleted MDA-MB-231 cells and DEHP-exposed clones were seeded at a density of 2x10^5^ cells/well in 6-well plates, incubated at 37 °C overnight, and then the medium was changed to serum-free medium, after which the cells were incubated until 85-95% confluence was reached. The culture medium was collected, and dead cells were removed by centrifugation at 1500 rpm at 4 °C for five minutes. The medium was lyophilized via a freeze dryer, and the proteins were reconstituted in 100 µl of 1X PBS. Proteins were then separated on a 2% gelatin gel and incubated in development buffer for 24 h at 37 °C. These gels were stained with Coomassie brilliant blue for 30 min while agitated. Furthermore, the gel was treated with destaining solution until clear bands were observed and photographed.

### Coimmunoprecipitation and peptide sequencing

MSI2-interacting proteins and subsequent signaling pathways were evaluated using coimmunoprecipitation (co-IP), LC/MS/MS, and peptide identification. A Dynabeads® Co-Immunoprecipitation Kit (Invitrogen, 14321D) was used for co-IP of MSI2-interacting proteins with an MSI2 antibody as bait. Briefly, the MSI2 antibody was conjugated to Dynabeads^®^ M-270 epoxy magnetic beads (7 µg/mg beads) according to the manufacturer's protocol. Fifty milligrams of parental MDA-MB-231 and DEHP-exposed clones (dry weight) were lysed with 1X IP buffer for 15 min on ice. The lysates were centrifuged, and the supernatants were used for the IP assay. Antibody-coupled epoxy Dynabeads® (1.5 mg/sample) were incubated with the cell lysates at 4 °C on a rotor, the beads were washed, and the proteins were eluted. The eluted proteins were lyophilized via a vacuum concentrator and then solubilized with loading buffer. These protein samples were resolved by SDS-PAGE, and the gels were subjected to Coomassie blue staining. The protein bands were cut and processed for protein isolation and identification.

These excised gels were commissioned to Biotools Co. (Taipei, Taiwan) for protein identification. Briefly, in-gel IP products were separated and reduced with DTT. Next, the samples were subjected to cysteine blocking with iodoacetamide (55 mM, IAM, Sigma) and digested with modified porcine trypsin (sequencing grade, Promega). The digested peptides were extracted from the gel, vacuum dried and dissolved in 0.5% formic acid for analysis via LC‒MS/MS. The LC apparatus was coupled with a 2D linear ion trap mass spectrometer (Orbitrap Elite ETD; Thermo Fisher) operated by Xcalibur v2.2 software (Thermo Fisher Scientific). The full-scan MS was conducted with the Orbitrap over a range of 400 to 2,000 Da and a resolution of 120,000 at m/z 400. Protein identification was further performed via data analysis via Proteome Discoverer Software (v1.4; Thermo Fisher). The MS/MS spectra were searched against the NCBI (RefSeq) database via Mascot (v2.5 Matrix Science, London, UK) via the online search engine http://www. matrixscience.com/.

### miRNA evaluation and quantification

To investigate the mechanism that MSI2 upregulation in DEHP-exposed MDA-MB-231 cells, we analyzed specific miRNAs that can target and influence MSI2 expression. Web-based miRNA target prediction databases such as miRTargetLink Human 24, TargetScanHuman (Release 7.2) 25, miRBD 26, and miRmap 27 were used to evaluate the ability of miRNAs predicted to target MSI2, their binding sites and their binding affinity.

Furthermore, to quantify the expression of the predicted miRNAs, total RNA was extracted via the TRIzol^TM^ reagent (Thermo Fisher, #10296010). miRNAs were reverse transcribed to complementary DNA via a Mir-X^TM^ miRNA First-Strand Synthesis Kit (Takara, #638313, Japan) based on the instructions of the manufacturer. SYBR Green PCR Master Mix and predesigned forward and reverse primers for miR-155-5p and U6-snRNA (Table 2) (Genomics, Taipei, Taiwan) were used to conduct the quantitative real-time polymerase chain reaction (qPCR) on a QuantStudio 5 Real-Time PCR system (Applied Biosystems^TM^, A34322). The changes in miRNA expression were calculated from Ct values and the equation 2^-ΔΔCt^, and the results were further analyzed by the software QuantStudio^TM^ Design & Analysis (v1.5x, Applied Biosystems^TM^), and SigmaPlot (v12.3, Systat Software Inc., Germany) was used for quantification.

### miRNA mimic transfection and effects on MSI2-mediated cell migration

To evaluate the effect of miR-155-5p on MSI2 expression, we transfected a miRNA mimic into DEHP-exposed clone #1 cells and evaluated its effect on MSI2 mRNA and protein expression. The miR-155-5p mimic (TJ-dsmim2OD) and its negative control mimic (TJ-dsmiNC) were obtained from Biotools Co. Ltd. (Taipei, Taiwan). A total of 1x10^6^ DEHP-exposed clone #1 cells were seeded in 6-well plates and incubated overnight before they were transfected with negative control mimic or miR-155-5p mimic in OptiMEM (Gibco, #31985062) via Lipofectamine 2000 (Invitrogen, #11668019) and incubated for 36 h at 37 °C. Following incubation, protein extraction was performed for western blotting, and total RNA was extracted via TRIzol^TM^ reagent (Thermo Fisher, #10296010) and reverse transcribed to cDNA via a high-capacity cDNA reverse transcription kit (Applied Biosystems, #4368814). qPCR was conducted with predesigned forward and reverse primers against MSI2, vimentin, and β-actin (Table 3) obtained from Genomics Co. Ltd. (Taipei, Taiwan) on a QuantStudio^TM^ 5 Real-Time PCR system (Applied Biosystems, A34322). The data were analyzed via the software QuantStudio^TM^ Design and Analysis (v1.5x, Applied Biosystems^TM^), and SigmaPlot was used for quantification.

The effect of miR-155-5p on the migration of DEHP-exposed clone #1 cells was assessed via a wound healing assay. A total of 1x10^5^ of DEHP-exposed clone #1 cells were seeded in a 12-well plate at 37 °C overnight. Afterwards, the cells were transfected with various concentrations of negative control mimic or miR-155-5p mimic for 36 h. A scratch was created when the transfected cells formed a monolayer, and cell migration across the wound was assessed for 20 h.

### Luciferase assay

To confirm the specificity of miR-155-5p for MSI2 mRNA, a luciferase reporter assay was conducted. The human MSI2 (NM-170721) 3′ UTR luciferase reporter clone was purchased from Origene Technologies, Inc., Rockville, USA. A total of 2.5 × 10^4^ 293T cells were seeded in a 96-well plate and incubated at 37 °C until approximately 80% confluence was achieved. After incubation, the cells were transfected with 10 ng of plasmid either alone or with the negative control mimic/miR-155-5p mimic via Lipofectamine 2000 (Invitrogen, #11668019) in OptiMEM (Gibco, #31985062) and incubated at 37 °C for 36 h. A dual-Glo^®^ Luciferase Assay System (Promega, #E2920) was used for the luciferase assay. Firefly luciferase activity was read via a luminometer, and the data were analyzed and quantified via SigmaPlot software.

### Analysis of proliferation markers

To evaluate the effect of MSI2 knockdown on MDA-MB-231 cell proliferation, qPCR and IF staining were performed. TRIzol^TM^ reagent (Thermo Fisher, #10296010) was used to extract RNA from scramble-treated and MSI2-depleted MDA-MB-231 cells. cDNA was synthesized via a high-capacity cDNA reverse transcription kit (Applied Biosystems, #4368814). To determine the changes in specific genes, a QuantStudio^TM^ 5 Real-Time PCR system (Applied Biosystems, #A34322) was used with predesigned forward and reverse primers against Ki67, PCNA, and β-actin (Table 4), which were synthesized by Genomics Co. The data were analyzed using QuantStudio^TM^ Design and Analysis (v1.5x, Applied Biosystems^TM^), and quantitative analysis was conducted via SigmaPlot software.

MDA-MB-231 cells (2.5x10^4^ scramble-treated or MSI2-knockdown cells) were seeded in cell chamber slides (SPL Lifesciences, #30114) and incubated for 24 h at 37 °C. Immunofluorescence was performed using antibodies against Ki67 and PCNA.

### *In vivo* metastasis and tumorigenesis

To evaluate and validate the role of MSI2 in tumorigenesis, especially the metastasis of long DEHP-exposed TNBC cells, an *in vivo* assessment of a xenograft tumor model was conducted. Female BALB/c nude mice (8 weeks old) were obtained from the National Experimental Animal Center (Taipei, Taiwan), and the maintenance and experimental procedures were performed with specific pathogen-free mice (Kaohsiung Medical University, Kaohsiung Medical University, Taiwan) following the guidelines and approval of the IACUC, Kaohsiung Medical University, Kaohsiung, Taiwan (Approval No. 108182).

These mice were divided into the following four groups: Scr Ctrl, shMSI2 Ctrl, Scr Clone #1, and shMSI2 Clone #1. Scramble-treated, MSI2-depleted MDA-MB-231 or DEHP-exposed clone #1 cells (1x10^5^) were injected into the fourth mammary fat pad of the mice via an incision in the abdomen (6 mice per mouse). The body weights and tumor volumes of the mice were measured twice a week, and 9 weeks later (the endpoint), the mice were sacrificed. Samples from tumor tissues and organs (lung, liver, kidney, mammary fat pad, and lymph node) were collected to assess distant metastasis. Formalin-fixed lung tissue samples were sent to Scientific Integration Design Service Corporation (SIDSCO, Kaohsiung, Taiwan) for immunohistochemical (IHC) and hematoxylin and eosin (HE) staining. Briefly, formalin-fixed tissues were embedded in paraffin and sectioned. Mammary fat pad and lymph node tissue sections were processed for HE staining to evaluate breast cancer metastasis. Lung tissues were processed for IHC staining to evaluate the expression of MSI2 and the metastasis markers vimentin, N-cadherin, and E-cadherin. Antibody against HLA class I ABC was used as marker for presence of human origin cells/human TNBC cells metastasis to mice lungs.

### *In vivo* imaging

*In vivo* breast cancer metastasis tracking was performed via a high-content IVIS spectrum imaging system (PerkinElmer, Waltham, MA, USA). Briefly, stable luciferase-expressing cells were established by transfecting cells with a luciferase-expressing plasmid (pLAS2w.FLuc. Ppuro) (RNAi Core Facility, Academia Sinica, Taipei, Taiwan) in untreated MDA-MB-231 and DEHP-exposed clone #1 (scramble-treated and MSI2-depleted) cells. A total of 5x10^5^ luciferase-expressing stable cells were transplanted into the mammary fat pads of 8-week-old female BALB/c nude mice. Tumor progression and metastasis were tracked by intraperitoneally injecting 200 μl of D-luciferase (15 mg/ml) (Gold Biotechnology, LUCK-1G) into the abdomen of anesthetized mice and using an imaging system to track luminescence.

### Statistical analysis

All the data were analyzed via SigmaPlot (v12.3, Systat Software Inc., Germany) and GraphPad Prism software (v9.0), and the results are presented as the means ± standard deviations. The clinical data were analyzed via SPSS software (v200, IBM-SPSS Inc., IL, USA). Comparisons between two groups were analyzed by Student's t test, while comparisons among multiple groups and the results of the *in vivo* assay were evaluated by one-way analysis of variance (ANOVA). A *p* value < 0.05 was considered statistically significant.

## Results

### Prolonged DEHP exposure facilitates migration and invasion in TNBC

**Figure 1:**
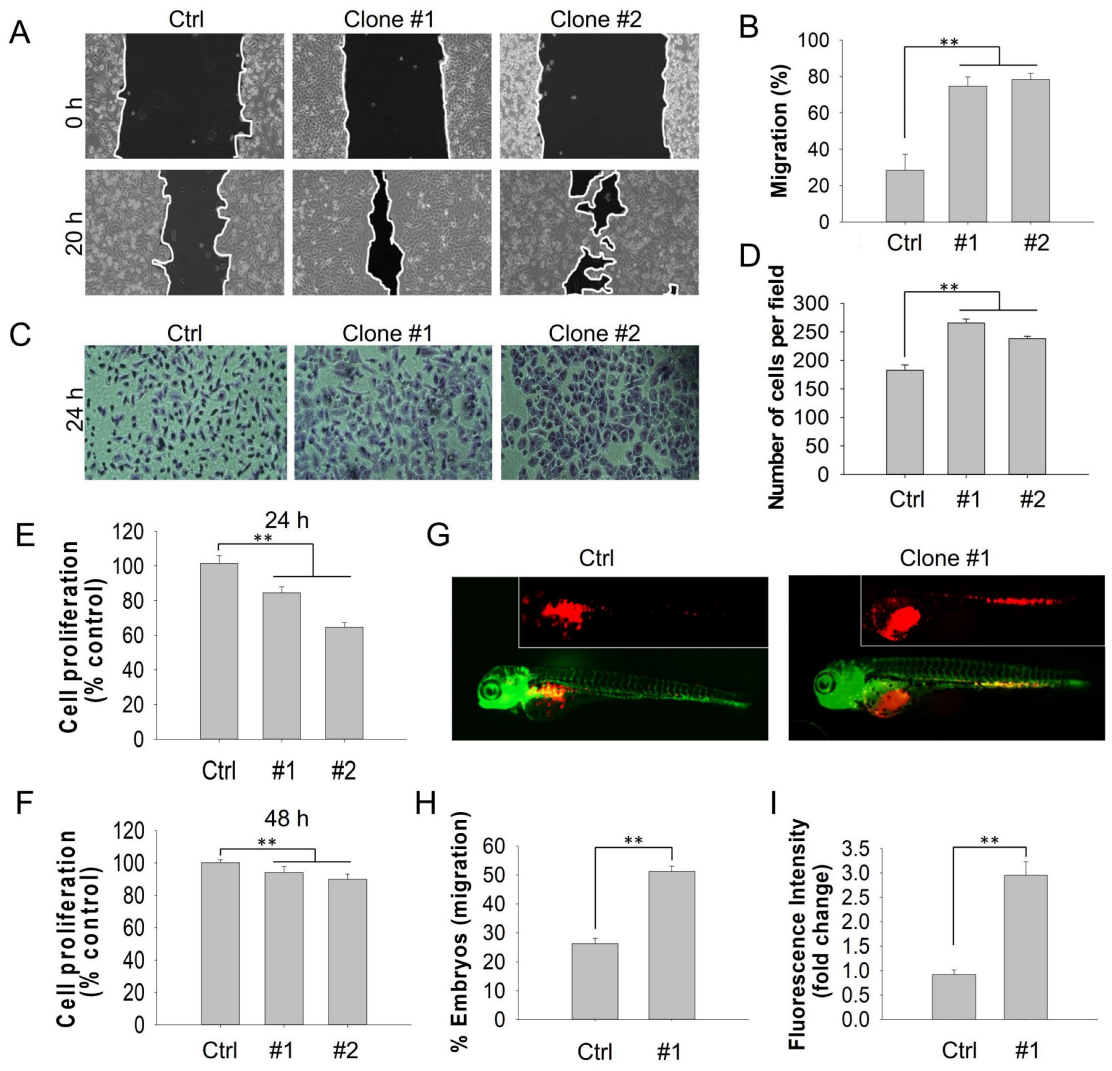
** Prolonged DEHP exposure enhanced the migration and invasion of MDA-MB-231 cells both *in vitro* and* in vivo***.** (A)** Cell migration of untreated and DEHP-exposed MDA-MB-231 cells evaluated by a wound healing assay for 20 h. **(B)** Quantitative analysis of cell migration (means ± SD). **(C)** Cell invasion of untreated and DEHP-exposed MDA-MB-231 cells were assessed with Matrigel-coated Transwell inserts for 24 h. **(D)** Quantitative analysis of cell invasion (means ± SD). **(E-F)** Cell proliferation evaluated by the MTT assay at 24 h and 48 h. **(G)** Zebrafish xenograft assay to confirm *in vivo* cell migration in Tg (*fli1:EGFP*) zebrafish embryos (fluorescence images obtained at 24 hpi). **(H)** Quantification of the percentage of zebrafish embryos showing cell migration to SIV (means ± SD, n=50). **(I)** Quantitative analysis of cell migration to the zebrafish SIV (fluorescence intensity indicative of cell number, fold change vs control); ** P < 0.001.

Emerging studies have demonstrated that DEHP exposure potentially promotes migration and invasion in several types of cancer. For instance, DEHP treatment enhances NSCLC progression through the activation of Wnt/β-catenin signaling 10. Moreover, Chen and colleagues suggested that DEHP and its metabolite mono(2-ehylhexyl) phthalate (MEHP) facilitated the migration and invasion of colon cancer cells 28. To determine the effect of long-term DEHP exposure on the migration and invasion of breast cancer cells, wound healing and cell invasion assays were performed. Compared with untreated MDA-MB-231 and Hs578T cells, DEHP-exposed MDA-MB-231 (clones #1 and #2) and Hs578T cells showed increased migratory capacity (Fig. 1A-B and S1A i-ii). However, no significant difference was observed between prolonged DEHP-exposed and untreated MCF7 cells and HMECs (Fig. S1B i-ii; Fig. S1C i-ii). Consistent with the migration assay results, DEHP-exposed MDA-MB-231 and Hs578T cells exhibited a remarkable increase in the number of invading Matrigel cells, compared with untreated MDA-MB-231 and Hs578T cells (Fig. 1C-D and Fig. S1A iii-iv). In contrast, no significant effect was observed in HMECs (Fig. S1C iii-iv). Interestingly, there was a decline in the number of invading DEHP-exposed MCF7 cells (Fig. S1B iii-iv). These findings indicate that long-term DEHP exposure promoted migration and invasion, specifically in TNBC cells*.* In addition, a cell proliferation assay was employed to determine whether DEHP-induced migration was mediated by proliferation. Long-term DEHP treatment inhibited the proliferation of DEHP-exposed MDA-MB-231 clones #1 and #2 at 24 h and 48 h (Fig. 1E-F). These results suggested that the increased migration of DEHP-exposed MDA-MB-231 cells was not affected by the proliferation rate.

On the basis of the *in vitro* results, we further validated whether the DEHP exposure-induced increase in migration ability was consistent *in vivo*. To this end, a zebrafish xenograft cell migration assay was performed. Compared with embryos injected with untreated cells, 48 hpf embryos injected with DEHP-exposed MDA-MB-231 (clone #1) and Hs578T cells presented increased cell migration from the embryo yolk sac to the SIV at 24 hpi (Fig. 1G and S1D i). Furthermore, more than 50% and 70% of the embryos injected with DEHP-exposed MDA-MB-231 (clone #1) and Hs578T cells, respectively, migrated to SIV, whereas 25% and 30% of the embryos injected with untreated MDA-MB-231 and Hs578T cells, respectively, migrated to SIV (Fig. 1H and Fig. S1D ii). Quantitative analysis of the number of cells that migrated to SIV revealed 3-fold and 1.4-fold greater cell migration (fluorescence intensity of DiI-stained cells) in embryos injected with DEHP-exposed MDA-MB-231 (clone #1) and Hs578T cells, respectively (Fig. 1I and Fig. S1D iii). Overall, long-term DEHP exposure potentially induces TNBC cell migration and invasion both *in vitro* and *in vivo*.

### MSI2: a predicted candidate for DEHP-mediated migration and invasion in MDA-MB-231 cells

**Figure 2:**
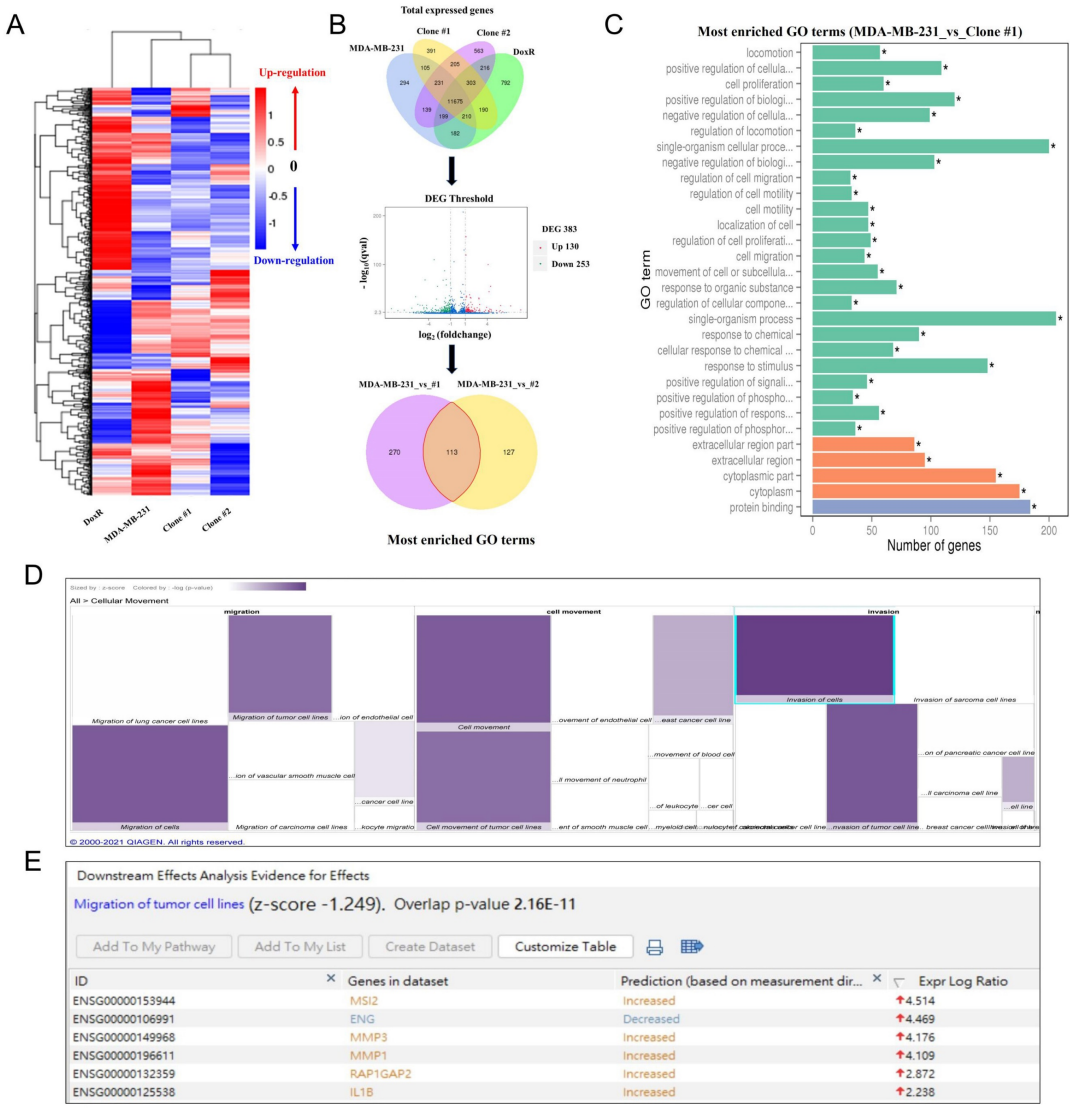
** Involvement of MSI2 in DEHP-induced migration and invasion as predicted by NGS analysis. (A)** Heatmap of DEGs in control and DEHP-exposed cells. **(B)** Venn diagram of total DEGs overlapping among different samples; volcano plot of the DEGs; pie chart of common differentially expressed GO terms in control and DEHP-exposed clones #1 and #2. **(C)** Bar chart of significantly enriched GO terms and the number of DEGs enriched in biological processes (green) and cellular components (orange); (* significant enrichment). **(D)** IPA-derived heatmap analysis of cellular movement under various conditions and the functions of the DEGs involved. **(E)** Downstream analysis of genes involved in the migration and invasion of tumor cell lines, highlighting increased MSI2 expression.

As demonstrated above, prolonged DEHP treatment facilitates the migration and invasion of TNBC cells; nevertheless, the underlying mechanism remains unclear. Hence, next-generation sequencing (NGS) was employed to identify the genes and signaling pathways involved in DEHP-induced migration and invasion of MDA-MB-231 cells. A heatmap of cluster analysis was used to determine gene expression patterns under different experimental conditions. By clustering genes with similar expression patterns, the unknown function of genes or the function of unknown genes could be recognized. In hierarchical clustering, areas with different colors represent different groups of clusters, and genes within each group might have similar functions or participate in the same biological process (Fig. 2A). A Venn diagram illustrating coexpression was created for the different experimental groups, such as untreated MDA-MB-231 cells and DoxR- and DEHP-exposed clones. Genes with significantly different expression among the groups were screened (default threshold of the fragments per kilobase of transcript per million mapped reads (FPKM) value =1) and then summarized to construct a Venn diagram. The coexpressed genes are visually presented as significantly differentially expressed genes among the different groups. Volcano plot showing the overall distribution of DEGs among MDA-MB-231 and DEHP-exposed clone #1 cells. The threshold is normally set as |log2(FoldChange)| > 1 and a q value < 0.005. The Venn diagram represents the number of DEGs in each group (MDA-MB-231 vs clone #1 and MDA-MB-231 vs clone #2) and the overlaps between groups. A total of 383 DEGs were reported in the MDA-MB-231 vs clone #1 comparison, and 240 genes were reported in the MDA-MB-231 vs clone #2 comparison. It was noteworthy that 113 common genes were identified in both groups (Fig. 2B).

The GO enrichment analysis results revealed the 30 most enriched terms along with the number of genes associated with the listed GO terms. Cellular locomotion, positive regulation of cellular motion, regulation of locomotion, and regulation of cell migration and motility were significantly enriched in the DEHP-exposed clone #1 (Fig. 2C). Apart from KEGG analysis, IPA was used for the validation and identification of the involved molecular pathways. The IPA of disease and function revealed a significant increase in cellular migration and movement, which was consistent with the GO results of the NGS data (Fig. 2D). Downstream analysis of genes involved in the migration of tumor cell lines revealed that MSI2 was the top regulator of migration, with a log ratio of 4.514, followed by MMP3, MMP1, and IL1β (Fig. 2E). Altogether, MSI2 is a predicted candidate that plays a crucial role in the DEHP exposure-induced migration of MDA-MB-231 cells.

### MSI2 depletion suppresses the migration and invasion of DEHP-exposed MDA-MB-231 cells *in vitro* and *in vivo*

**Figure 3:**
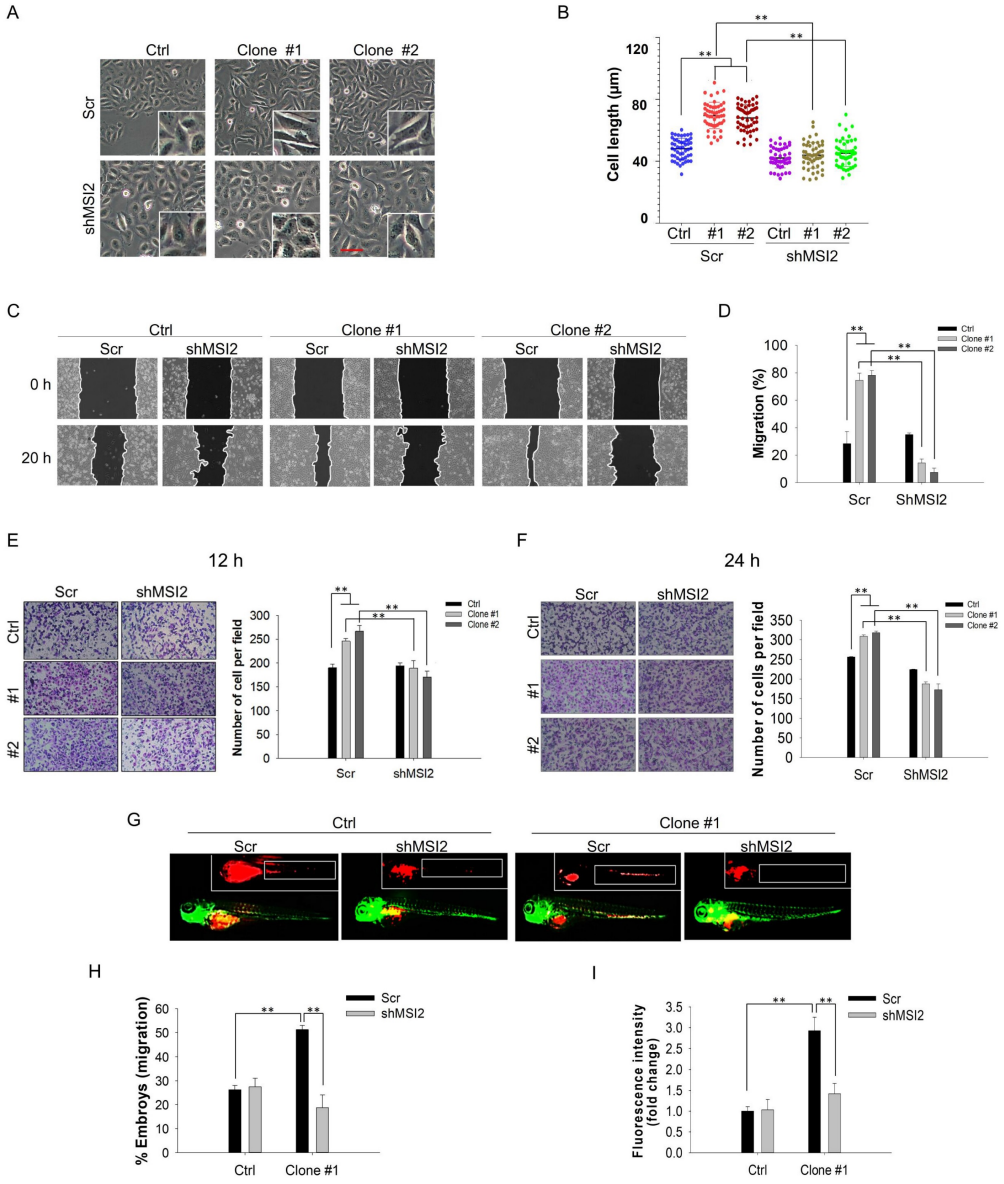
** MSI2 knockdown reversed DEHP-induced migration and invasion *in vitro* and *in vivo*. (A)** Cell morphology of Scr-treated and MSI2-silenced MDA-MB-231 and DEHP-exposed clones as observed by light microscopy. Scale bar = 150 µm. **(B)** Quantitative analysis of cell length (means ±SDs). **(C)** A wound healing assay was performed to validate the role of MSI2 in DEHP-induced migration in Scr- and MSI2-silenced MDA-MB-231 cells and in DEHP-exposed clones. **(D)** Quantitative analysis of cell migration at 20 h after wound formation (mean ± SD). (E-F) Matrigel-coated Transwell assay to evaluate the effect of MSI2 knockdown on cell invasion and quantitative analysis of cell invasion for (E) 12h and (F) 24h. **(G)** Zebrafish xenograft assay to evaluate the cell migration of Scr-treated and MSI2-silenced MDA-MB-231- and DEHP-exposed clones in 48 hpf Tg (*fli1:EGFP*) zebrafish embryos (fluorescence image captured at 24 hpi). **(H-I)** Quantification of embryos showing metastasis to SIV (mean ±SD, n=50) and fluorescence intensity analysis of cells that migrated to SIV in zebrafish embryos (fluorescence intensity reflects the cell number, fold change vs. control); ** P < 0.001.

**Figure 4:**
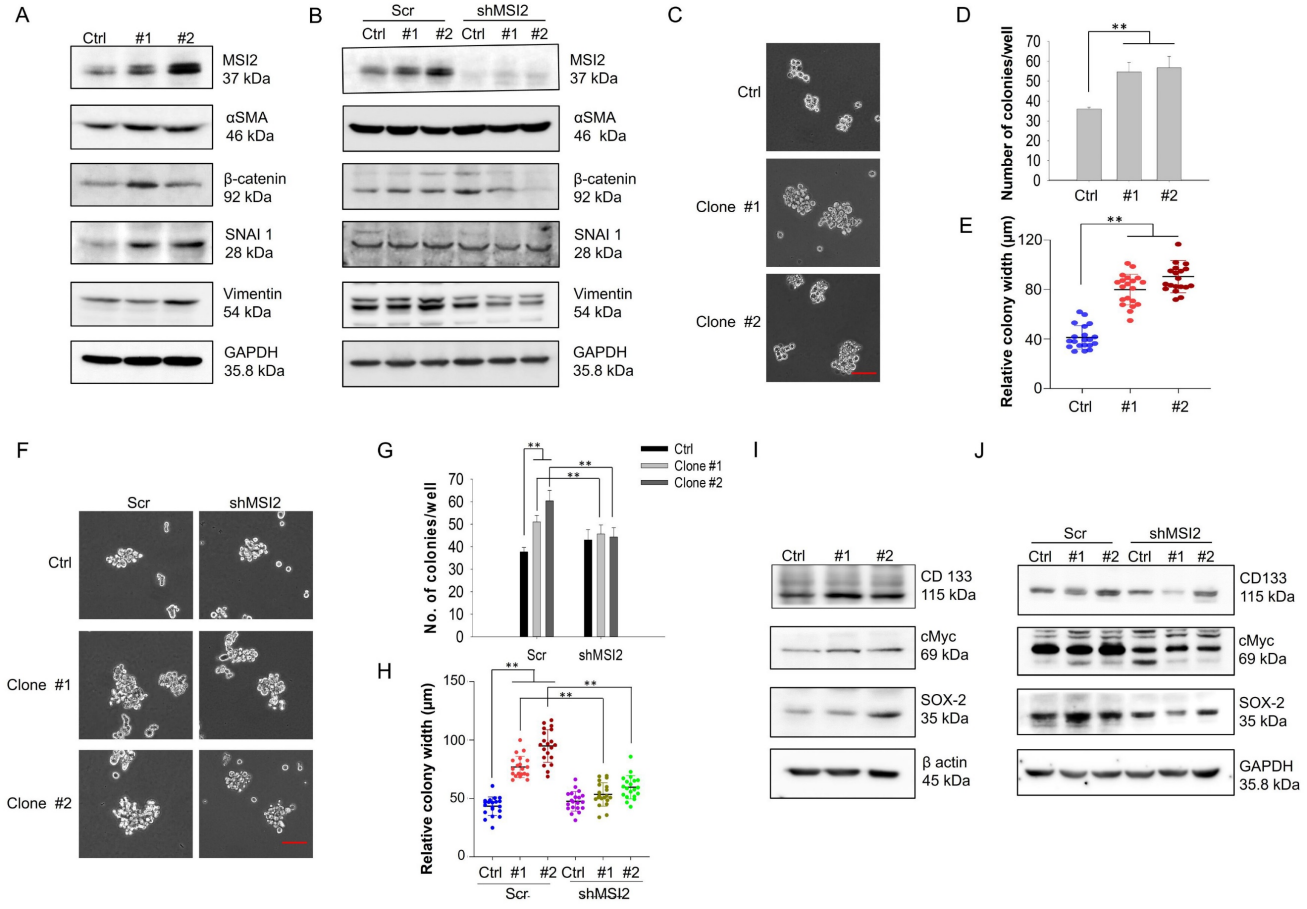
** Prolonged DEHP exposure induces EMT and stemness in MDA-MB-231 cells in an MSI2-dependent manner. (A)** Changes in the expression of the EMT markers α-SMA, β-catenin, SNAI1, and vimentin, as evaluated by western blotting. **(B)** Evaluation of the effect of MSI2 knockdown on the expression of EMT markers by western blotting.** (C)** Anchorage-independent growth/spheroid formation as evaluated by the soft agar colony formation assay. Scale bar = 50 µm. **(D-E)** Quantitative analysis of the number and size of colonies/spheroids originating from untreated and DEHP-exposed MDA-MB-231 cells.** (F)** Assessment of anchorage-independent growth/spheroid formation by soft agar colony formation assays in Scr-treated and MSI2-depleted cells. Scale bar = 50 µm. **(G-H)** Quantitative analysis of colony number and colony size in the soft agar colony formation assay (mean ± SD). **(I)** Changes in the expression of the stemness-related markers CD133, cMyc, and SOX-2 in untreated and DEHP-exposed clones evaluated by western blotting. **(J)** Evaluation of changes in the expression of stemness-related markers in Scr-treated and MSI2-depleted MDA-MB-231 cells and in DEHP-exposed clones; **P < 0.001.

Consistent with the NGS analysis results, MSI2 was upregulated in DEHP-exposed MDA-MB-231 clones #1 and #2 (Fig. 4B). To validate the oncogenic role of MSI2 in DEHP-induced cell migration and invasion, lentiviral knockdown of MSI2 was successfully established (Fig. 4B). First, morphological changes were observed since cells with increased migratory and metastatic capacities generally possess a spindle-shaped morphology along with increased cellular length 29. The morphology of the DEHP-exposed clones was mesenchymal-like, with elongation and spindle formation at the periphery of the cells (Fig. 3A). Moreover, the length of DEHP-exposed clones was greater than that of untreated MDA-MB-231 cells (Fig. 3B). Conversely, elongated cell morphology and cell length were reduced once MSI2 was depleted (Fig. 3A-B). Furthermore, we investigated whether MSI2 plays a crucial role in DEHP-induced TNBC migration and invasion. Cell migration was significantly inhibited in MSI2-knockdown DEHP-exposed clones (Fig. 3C-D). However, no significant effect was observed in untreated MDA-MB-231 cells. In the cell invasion assay, MSI2 depletion reduced the invasion capacity of DEHP-exposed clones, with MSI2-knockdown untreated MDA-MB-231 cells showing only a slight decrease in invasion capacity (Fig. 3E-F).

The results of the zebrafish xenograft metastasis experiments were consistent with those of the *in vitro* experiments. Embryos injected with scramble (Scr) shRNA-treated or MSI2-depleted MDA-MB-231 cells showed no change in cell migration. However, compared with embryos injected with DEHP-exposed clone #1, embryos injected with MSI2-knockdown DEHP-exposed clone #1 exhibited significantly reduced cell migration, as observed in the fluorescence images (Fig. 3G). In addition, the number of embryos with positive cell migration was markedly lower in the MSI2-depleted clone #1 embryos than in the Scr-treated clone #1 embryos (Fig. 3H). Fluorescence analysis, which reflects the quantity of migrated cells, revealed a significant reduction in the fluorescence intensity in MSI2-knockdown clone #1 embryos compared with Scr-treated clone #1 embryos (Fig. 3I). Overall, these results suggest that long-term DEHP exposure facilitates TNBC migration and invasion in an MSI2-dependent manner.

### Long-term DEHP exposure promotes EMT and cancer stemness in MDA-MB-231 cells via MSI2 overexpression

EMT is a biological process where epithelial cells acquire mesenchymal-like characteristics to facilitate invasion and metastasis 30. Additionally, EMT is important in the initial steps of metastasis 31; thus, we evaluated whether DEHP-induced MSI2 overexpression upregulated the expression of EMT-related markers. Our results revealed increased expression of EMT markers, including β-catenin, α-SMA, SNAI1, and vimentin, in DEHP-exposed clones (Fig. 4A), whereas β-catenin and vimentin were obviously downregulated after MSI2 knockdown. However, the expression of β-catenin and SNAI1 was slightly elevated in MSI2-knockdown MDA-MB-231 cells compared with Scr-treated cells (Fig. 4B). Given that EMT is closely associated with stemness 32 and that MSI2 has been reported to facilitate the stemness of liver cancer stem cells 33, 34, we determined whether prolonged DEHP exposure also confers TNBC cell stem-like properties. To this end, soft agar colony formation assays were performed, and the protein levels of stemness-related markers were evaluated. A soft agar colony formation assay revealed increased proliferation, resulting in the formation of larger spheroids from DEHP-exposed clones (Fig. 4C). The number and width of colonies/spheroids formed from DEHP-exposed clones were significantly greater than those formed from untreated MDA-MB-231 cells (Fig. 4D-E). However, MSI2 knockdown reduced the number and size of spheroids derived from DEHP-exposed clones (Fig. 4F-H). At the molecular level, prolonged DEHP treatment promoted the upregulation of CD133, cMyc, and SOX-2, whereas these stemness markers were inhibited following MSI2 knockdown (Fig. 4I-J). Altogether, these findings indicate that long-term DEHP exposure induces EMT and stemness in TNBC via MSI2 overexpression.

### Long-term DEHP exposure activates the MSI2-mediated PI3K/Akt/NFκB signaling axis in MDA-MB-231 cells

**Figure 5:**
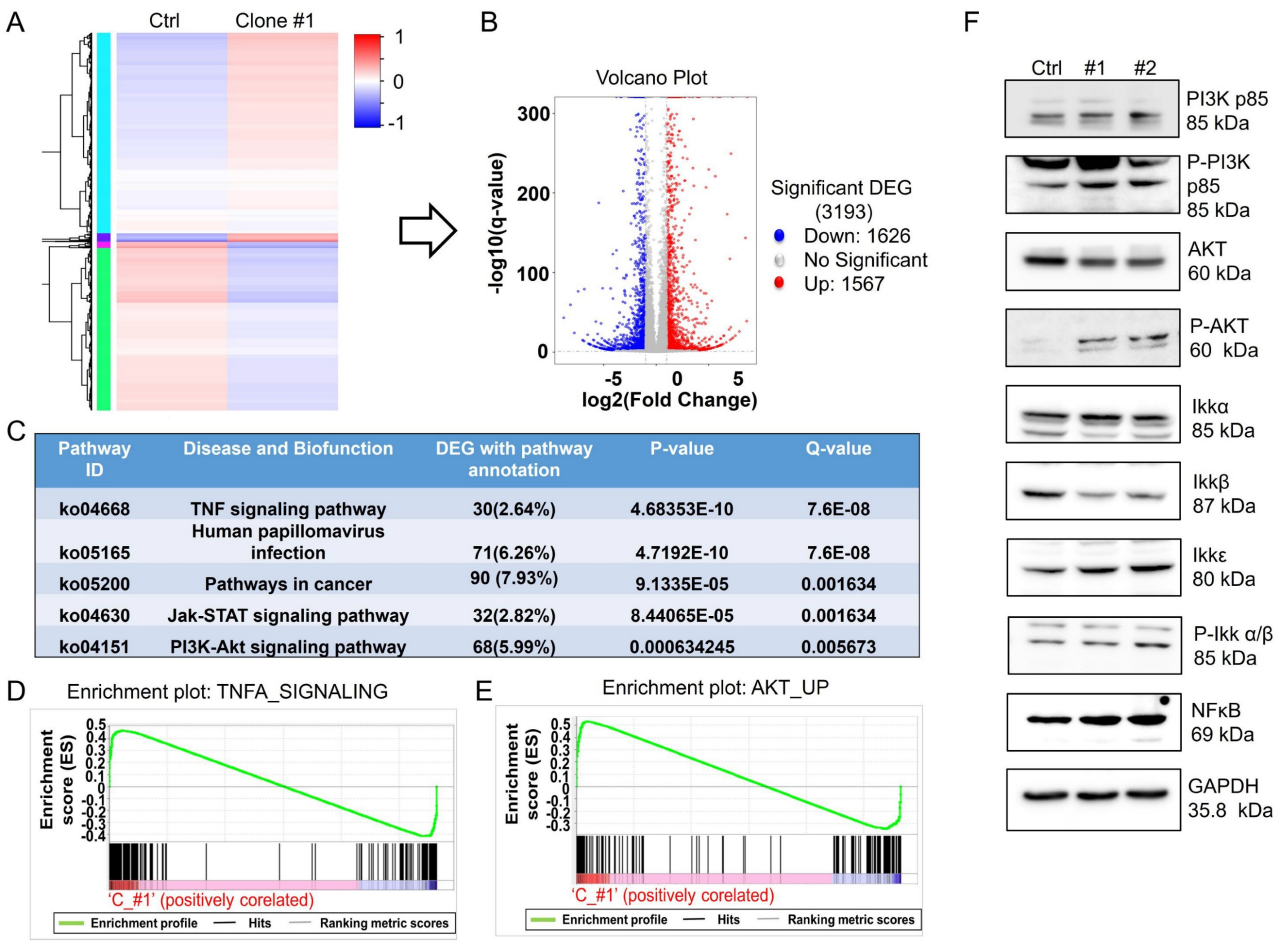
** MSI2 regulates the PI3K/Akt/NFκB signaling axis. (A)** Heatmap of DEGs from the co-IP protein complexes in control and DEHP-exposed cells. **(B)** Volcano plot of total DEGs in control and DEHP-exposed cells (red: upregulated; green: downregulated). **(C)** KEGG pathway analysis identified the 5 most significantly enriched pathways with DEG annotations, p values, and q values. **(D-E)** GSEA of TNFα and Akt signaling revealed a positive correlation in untreated MDA-MB-231 cells and DEHP-exposed clone #1 cells (enrichment score). **(F)** Evaluation of the expression of the PI3K/Akt/NFκB signaling markers PI3K p85, p-PI3K, Akt, p-Akt, Ikkα, Ikkβ, Ikkε, p-Ikkα/β, and NFκB in untreated MDA-MB-231 cells and DEHP-exposed clones.

MSI2, belonging to the Musashi RNA-binding protein family, is reportedly involved in posttranscriptional modification through binding with specific target mRNAs to regulate gene expression 35. To evaluate the potential interactions of RNAs with MSI2 and the role of MSI2 in the regulation of downstream signaling, we performed RNA-IP using an MSI2 antibody as bait to capture MSI2-RNA complexes. Total RNA pulled down from untreated MDA-MB-231 cells and DEHP-exposed clone #1 cells was sequenced for analysis of differential expression. A heatmap of cluster analysis results was generated to identify differential gene expression patterns between untreated MDA-MB-231 cells and DEHP-exposed clone #1 cells. The genes were clustered with similar expression patterns (hierarchical clustering), and genes within each group had similar functions or were involved in the same biological process (Fig. 5A). A volcano plot of the DEGs between DEHP-exposed clone #1 and untreated MDA-MB-231 cells was generated by plotting the statistical significance of the DEGs [log2 (fold change)] against genes expressed in the samples [-log10(q value); where the q value ≤ 0.05]. A total of 3193 genes were plotted, of which 1626 were downregulated and 1567 were significantly upregulated (Fig. 5B). KEGG enrichment analysis revealed that the TNF signaling, Jak-STAT signaling, and PI3K-Akt signaling pathways were the most significantly enriched pathways (Fig. 5C). The IPA of the NGS data revealed enrichment of genes related to cell movement and cell migration (Fig. S2A). Pathway enrichment analysis by IPA revealed that Jak-mediated activation of PI3K/Akt signaling regulated the expression of the transcription factor NFκB (Fig. S2B). Furthermore, gene set enrichment analysis (GSEA) of the DEGs revealed a positive correlation between TNFα signaling and Akt signaling in DHEP-exposed clone #1 (Fig. 5D-E). TNFα signaling was most significantly enriched; thus, the mRNA and protein levels of TNFα were determined. The results revealed no change in the mRNA or protein expression of TNFα between untreated MDA-MB-231 cells and DEHP-exposed clones (Fig. S3A-B). Furthermore, to validate the NGS and GSEA results, immunoblotting for the expression of PI3K/AKT/Ikk/NFκB signaling markers was performed. The upregulation of PI3K p85, phospho-PI3K p85, phospho-Akt, Ikkα, Ikkε, phospho-Ikk α/β, and NFκB p65 was observed in DEHP-exposed clones, indicating DEHP-induced activation of PI3K/Akt-mediated NFκB signaling (Fig. 5F). Surprisingly, the expression of Akt and Ikkβ was downregulated in DEHP-exposed clones. Taken together, these findings indicate that MSI2 mediates the activation of the PI3K/Akt/NFκB axis, which might play a vital role in DEHP-induced migration and invasion of MDA-MB-231 cells.

### NFκB mediated the transcriptional upregulation of MMP9 and cell migration in DEHP-exposed MDA-MB-231 cells

**Figure 6:**
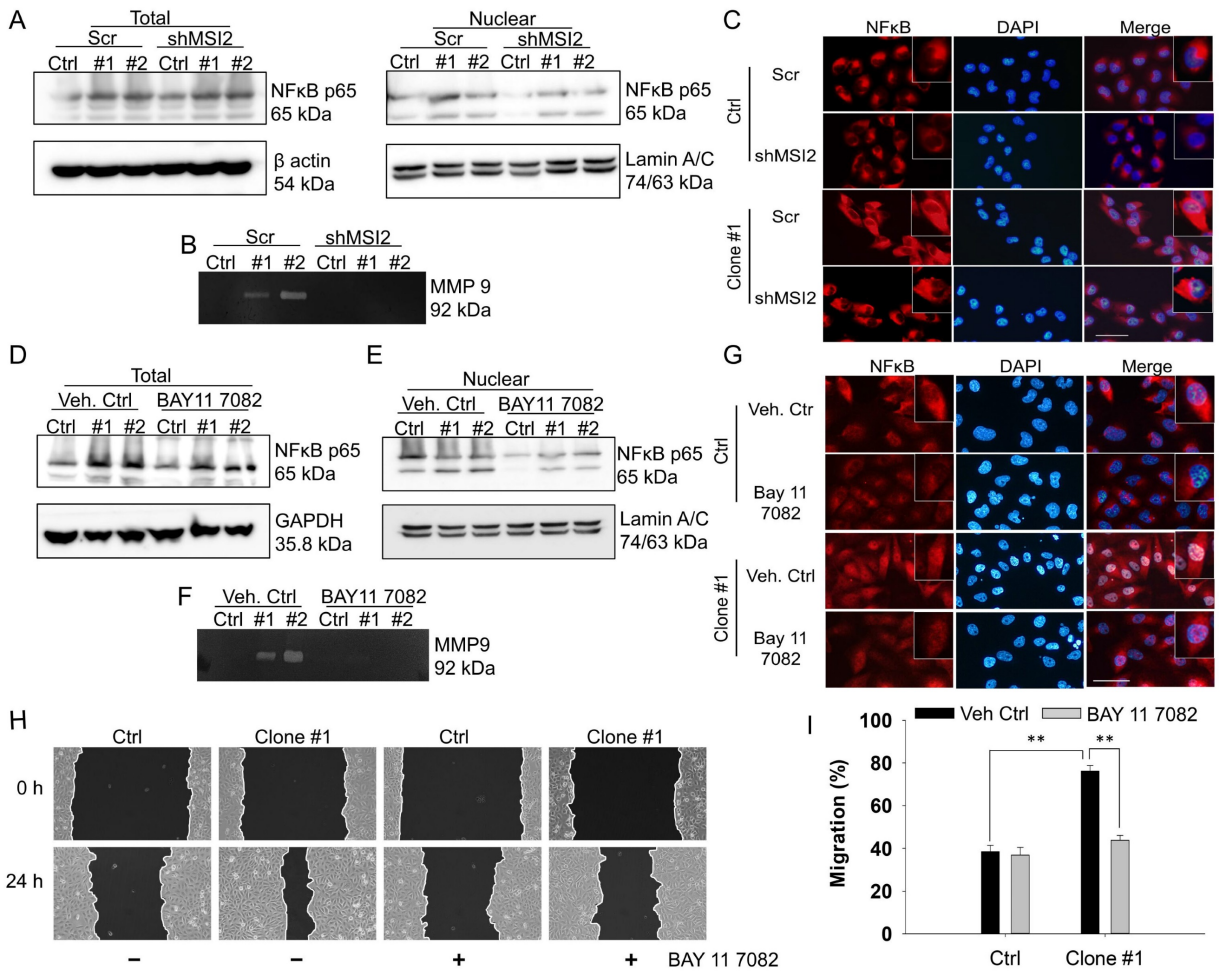
** NFκB controls DEHP-induced cell migration via MMP-9 regulation. (A)** Total and nuclear NFκB p65 expression levels in Scr-treated and MSI2-depleted MDA-MB-231 and DEHP-exposed clones were evaluated by western blotting. **(B)** Gelatin zymography analysis of MMP-9 expression in Scr-treated and MSI2-depleted MDA-MB-231 cells and DEHP-exposed clones. **(C)** IF analysis of intracellular NFκB p65 (red) localization and expression. Nuclear staining (blue). Scale bar = 100 µm. **(D-E)** Evaluation of total and nuclear NFκB p65 expression levels following BAY 11--7082 treatment (10 µM, 24 h) by western blotting in MDA-MB-231 and DEHP-exposed clones. **(F)** MMP-9 expression/activity analysis by gelatin zymography in BAY 11-7082 (10 µM, 24 h)-treated MDA-MB-231 cells and DEHP-exposed clones. **(G)** IF analysis of intracellular NFκB p65 (red) localization and expression following BAY 11--7082 (10 µM, 24 h) treatment. Nuclear staining (blue). Scale bar = 100 µm. **(H)** Effect of BAY 11-7082 (10 µM, 24 h) treatment on cell migration as evaluated by a wound healing assay for 24 h. **(I)** Quantitative analysis of cell migration at 24 h after wound formation (mean ± SD); **P < 0.001.

On the basis of the results of RNA-IP/NGS and IPA, we hypothesized that NFκB is involved in the regulation of MMPs. Indeed, an association between NF-κB signaling and the MMP9-mediated invasiveness of breast cancer has been previously reported 36, 37. Thus, the involvement of NFκB as a transcription factor of MMPs was evaluated via western blotting of total and nuclear proteins along with gelatin zymography. The western blotting results showed increased expression of total and nuclear NFκB in the DEHP-exposed clones. However, MSI2 depletion reduced the amount of NFκB in the nuclear fraction of clones #1 and #2 (Fig. 6A). Furthermore, gelatin zymography was performed to assess the activity of MMPs, and increased MMP9 activity was observed in DEHP-exposed clones. Similarly, MSI2 knockdown resulted in a decrease in MMP9 activity in clones #1 and #2 (Fig. 6B). To confirm the activation and nuclear localization of NFκB, IF staining was performed, the results of which revealed increased nuclear localization of NFκB p65 in clone #1, indicating the transcriptional activity of NFκB p65. MSI2 knockdown reduced the nuclear localization of NFκB p65 in MDA-MB-231 cells and DEHP-exposed clone #1 cells (Fig. 6C and Fig. S4A).

The NFκB inhibitor BAY 11-7082 was also used to confirm the role of NFκB in MMP9 transcriptional regulation and cell migration in DEHP-exposed MDA-MB-231 cells. No significant change in total NFκB p65 levels was observed in DEHP-exposed clones or untreated MDA-MB-231 cells (Fig. 6D). BAY11-7082 treatment downregulated nuclear NFκB p65 in MDA-MB-231 cells and DEHP-exposed clones (Fig. 6E). Moreover, BAY 11-7082 administration resulted in the loss of MMP9 activity, which confirmed that NFκB p65 is a transcriptional regulator of MMP9 (Fig. 6F). IF staining was performed to evaluate the intracellular localization of NFκB p65. Consistent with the western blotting results, IF showed reduced expression of NFκB p65 in the cytoplasm along with the loss of its nuclear localization in DEHP-exposed clone #1 (Fig. 6G and Fig. S4B). As expected, BAY 11-7082 treatment significantly reversed the migration of DEHP-exposed clone #1 cells, which verified the involvement of NF-κB signaling in DEHP-induced cell migration (Fig. 6H-I). These findings suggest that NFκB p65-mediated MMP9 expression is involved in the DEHP-induced migration of MDA-MB-231 cells.

### The novel MSI2-vimentin interaction potentially drives EMT in DEHP-exposed MDA-MB-231 cells

**Figure 7:**
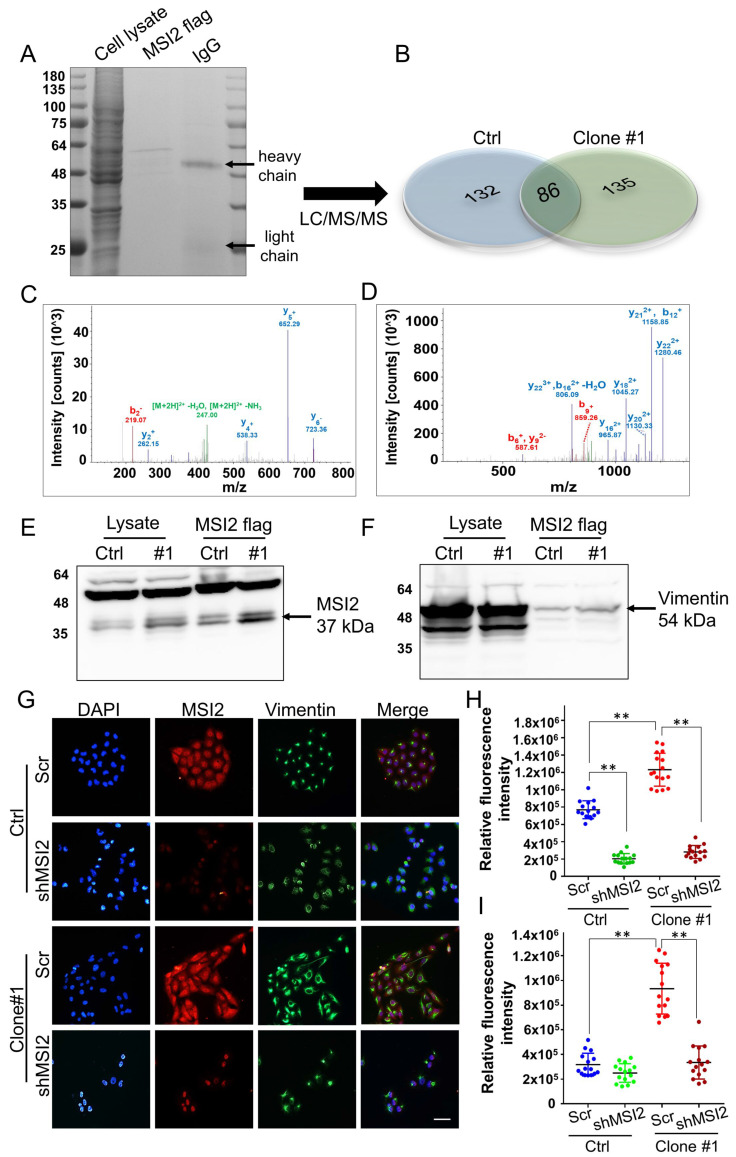
** MSI2 interacts with vimentin and regulates its expression and subcellular distribution. (A)** SDS-PAGE analysis of total cell lysates, Flag-MSI2 co-IP eluates, and antibodies (IgG). **(B)** LC/MS/MS analysis and protein identification evaluation (unique and shared proteins) of Flag-MSI2 co-IP protein complexes in untreated MDA-MB-231 and DEHP-exposed clone #1 cells performed by Proteome Discoverer software. **(C)** Unique peptide identification of LC/MS/MS vimentin (FANYIDK) and **(D)** MSI2 (IFVGGLSANTVVEDVKQYFEQFGK). x-axis: mass/charge ratio (m/z), y-axis: intensity of peak [count]. **(E-F)** Evaluation of MSI2 and vimentin coexpression in cell lysates and co-IP products by western blotting. **(G)** IF analysis of the intracellular localization and expression of MSI2 (red) and vimentin (green). Nuclear staining (blue). Scale bar = 50 µm. **(H-I)** Quantitative analysis of MSI2 (H) and vimentin (I) expression in Scr-treated and MSI2-depleted MDA-MB-231 cells and DEHP-exposed clone #1 cells (means ± SDs); **P < 0.001.

To identify potential MSI2-interacting proteins and further evaluate their roles in DEHP-induced migration of MDA-MB-231 cells, co-IP and mass spectrometry were performed. First, MSI2-interacting proteins were pulled down using an MSI2 antibody as bait, and protein complexes were digested and separated by SDS-PAGE along with total protein eluate (loading control) and antibody (Fig. 7A). Peptide isolation and identification revealed 132 and 135 unique proteins that interact with MSI2 in untreated MDA-MB-231 cells and in DEHP-exposed clone #1, respectively; 86 proteins were identified in both cell lines (Fig. 7B). Notably, vimentin was found to be the most abundant of the 86 proteins in both samples. Protein identification based on peptide sequencing revealed scores of 1088.30 and 5762.42 for vimentin in untreated MDA-MB-231 and DEHP-exposed clone #1, respectively (Table S1). The relative abundance of vimentin among the total identified shared proteins was 2.1% and 14.7% in untreated MDA-MB-231 cells and DEHP-exposed clone #1, respectively (Table S1). The LC‒MS/MS spectra of the unique 'FANYIDK' peptide of vimentin (Fig. 7C) and 'IFVGGLSANTVVEDVKQYFEQFGK' for MSI2 were identified (Fig. 7D). The results showed a strong interaction between MSI2 and vimentin. We then performed western blotting of protein complexes from co-IP experiments to confirm the results of the LC/MS/MS analysis. As expected, MSI2 and vimentin were upregulated in DEHP-exposed clone #1 samples subjected to Flag-MSI2 pulldown (Fig. 7E-F).

IF imaging was performed to evaluate the colocalization of MSI2 and vimentin. Strong MSI2 and vimentin expression was observed in DEHP-exposed clone #1 cells relative to untreated MDA-MB-231 cells, which is consistent with the western blotting results. MSI2 knockdown significantly reduced MSI2 expression in clone #1 and MDA-MB-231 cells. Vimentin was localized within the nuclear periphery in Scr-treated MDA-MB-231 cells, whereas strong vimentin expression was observed in the cytoplasmic region (vimentin filaments) of DEHP-exposed clone #1 cells. MSI2 knockdown in DEHP-exposed clone #1 cells reduced vimentin expression and colocalization in the perinuclear space, similar to what was observed in MSI2-silenced MDA-MB-231 cells (Fig. 7 G-I). MSI2 and vimentin were clearly colocalized in the cytoplasm. MSI2 knockdown reduced not only the expression of vimentin but also its distribution in the perinuclear space in DEHP-exposed clone #1. Furthermore, the vimentin filament distribution in the wound healing assay revealed the polarization of vimentin filaments in cells at the wound front in DEHP-exposed clone #1. However, in untreated MDA-MB-231 cells, a similar vimentin filament distribution was observed (Fig. S5). Taken together, these results are indicative of MSI2-mediated regulation and localization of vimentin; increased vimentin filament polarization corresponds to increased cell motility.

### miR-155-5p negatively regulates MSI2-mediated TNBC migration

**Figure 8:**
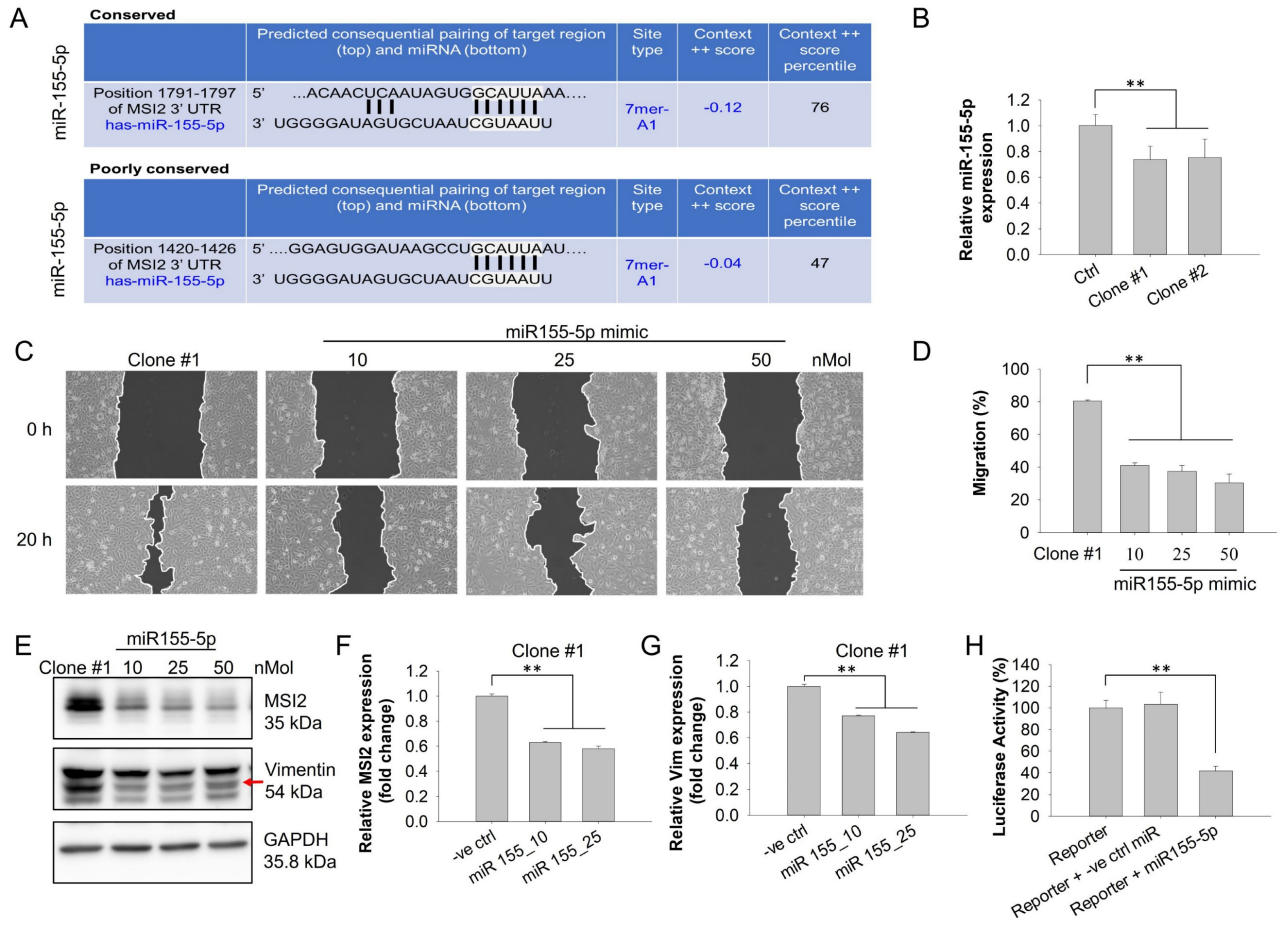
** miR-155-5p negatively regulates MSI2 expression and MSI2-induced migration. (A)** Evaluation of prediction-based MSI2-targeting miRNAs via the TargetScanHuman (Release 7.2) and miRBD miRNA target prediction databases. Conserved sites, site length and predicted position of the miR-155-5p binding site in the MSI2 3′UTR. **(B)** Evaluation of miR-155-5p levels by qPCR in untreated MDA-MB-231 cells and DEHP-exposed clones. **(C)** Effects of miR-155-5p mimic treatment (10, 25, or 50 nM) on cell migration, as evaluated by a wound healing assay 20 h after scratching. **(D)** Quantitative analysis of cell migration at 20 h after wound formation (means ± SDsSDs). **(E)** Evaluation of changes in the expression of MSI2 and vimentin in miR-155-5p mimic-treated (10, 25, 50 nMol) DEHP-exposed clones. **(F-G)** Evaluation of the effect of the miR-155-5p mimic on MSI2 (F) and vimentin (G) mRNA levels in DEHP-exposed clone #1 cells (means ± SDsSD). **(H)** Evaluation of miR-155-5p specificity with respect to the MSI2 3′UTR via a luciferase reporter assay via MSI2 3′UTR luciferase expression clone transfection in 293T cells (mean ±SD); **P < 0.001.

miRNAs are single-stranded noncoding RNAs (ncRNAs) 18-25 nucleotides in length that target one or more specific mRNAs to facilitate gene silencing 38, 39. Dysregulated miRNAs have been suggested to result in tumorigenesis, such as proliferation, resistance to apoptosis, angiogenesis, and metastasis 40. For example, miR-590-3p potently suppressed the lung metastasis of TNBC through downregulating slug 41. On the basis of these findings, we further evaluated whether altered MSI2 expression in DEHP-exposed clones was modulated by miRNAs. Hence, we performed miRNA target prediction via web-based miRNA‒mRNA target prediction databases. The miRTargetLink Human database was used to conduct gene-based miRNA prediction. The interaction networks generated by miRTargetLink contain experimentally validated interactions present in the latest version of miRTarBase (Release 6.0: Sept. 15, 2015) as well as from in-house generated data. The results of the analysis revealed a strong interaction between the MSI2 gene and miR-155-5p. To confirm the prediction results of miRTargetLink, we conducted a prediction analysis with the databases TargetScanHuman (Release 7.2) and miRBD. The prediction results revealed that the 3′UTR of MSI2 mRNA contains two conserved 7-mer-A1 sites for the miR-155-5p seed sequence. The first conserved site was positioned at 1791--1797 of the MSI2 3′UTR with a context score percentile of 76, and the second, albeit poorly, conserved site was positioned at 1420--1426 of the MSI2 3′UTR with a percentile score of 47 (Fig. 8A). Overall, web-based prediction suggested a strong interaction between MSI2 and miR-155-5p.

First, the relative expression of miR-155-5p was evaluated via qPCR, which revealed that compared with untreated MDA-MB-231 cells, DEHP-exposed clones presented lower expression of miR-155-5p (Fig. 8B). This result suggested that miR-155-5p might downregulate MSI2 expression. A wound healing assay revealed that miR-155-5p mimic treatment inhibited the migration of DEHP-exposed clone #1 cells in a dose-dependent manner (Fig. 8C-D). Additionally, both the mRNA and protein levels of MSI2 and vimentin were significantly reduced following miR-155-5p mimic transfection (Fig. 8E-G). To further confirm the regulatory effect of miR-155-5p on MSI2, a luciferase reporter assay was performed to validate the specificity of the interaction between miR-155-5p and MSI2 mRNA. The results demonstrated that luciferase activity was decreased in the cells transfected with the miR-155-5p mimic (Fig. 8H). This finding indicated a specific interaction between the 3′UTR of MSI2 mRNA and miR-155-5p. Hence, prolonged DEHP exposure likely induces the migration of TNBC cells by regulating the miR-155-5p/MSI2/vimentin axis.

### MSI2 facilitates TNBC metastasis but not proliferation *in vivo*

**Figure 9:**
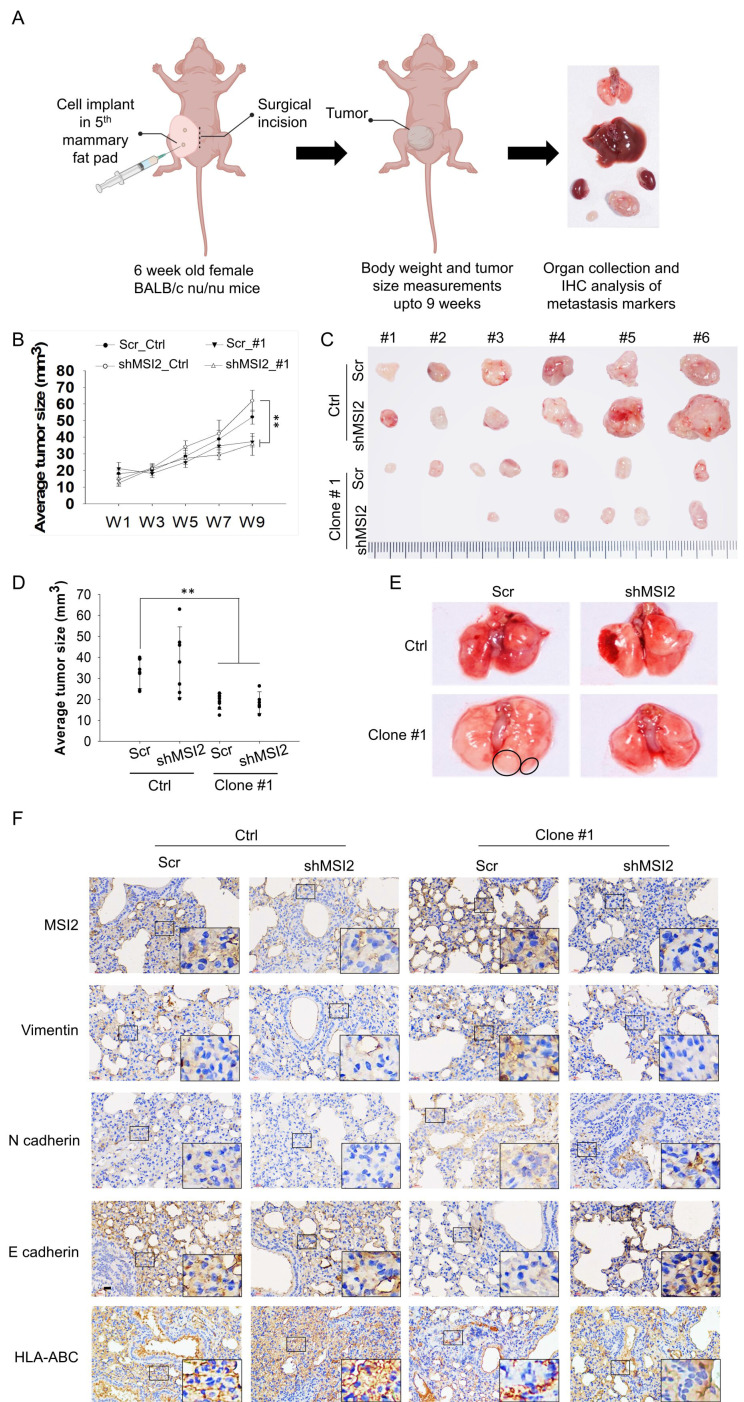
** MSI2 knockdown reduces DEHP-induced breast cancer metastasis *in vivo*. (A)** The effect of prolonged DEHP treatment on TNBC cell metastasis was evaluated in a mouse metastasis model. Untreated and DEHP-exposed MDA-MB-231 cells (Scr and MSI2 knockdown) were implanted into the mammary fat pads of 8-week-old female BALB/c nude mice. The average tumor size was recorded. The mice were sacrificed and processed for evaluation of metastasis. **(B)** Evaluation of average tumor sizes in different groups (mean ±SD). **(C-D)** Evaluation of tumor growth and size measurements of excised tumors (means ± SDs).** (E)** Evaluation of lung metastasis by morphological changes and changes in the size of the excised lungs. **(F)** Lung metastasis evaluation via IHC analysis of the metastasis-associated markers vimentin, N-cadherin, E-cadherin, and HLA-ABC. Scale bar = 30 µm. *P < 0.05, **P < 0.001.

To validate the results of the *in vitro* study and zebrafish xenograft model, we established a metastatic mouse model. MDA-MB-231- and DEHP-exposed clone #1 (Scr-treated and MSI2-knockdown) cells were implanted into the mammary fat pads of 8-week-old female BALB/c nude mice. Tumor growth was recorded for 9 weeks, at which point the mice were sacrificed to collect body tissues for metastasis evaluation (a schematic diagram is shown in Fig. 9A). The tumor load/volume was recorded weekly with calipers. Consistent with the *in vitro* cell proliferation results, we observed a relatively smaller average tumor mass in the Scr-treated clone #1 tumor-bearing group than in the Scr-treated MDA-MB-231 tumor-bearing group. MSI2 knockdown in clone #1 did not affect tumor size, indicating that MSI2 might not be related to cell proliferation in DEHP-exposed cells. Intriguingly, mice with tumors derived from MSI2-silenced MDA-MB-231 cells presented significantly greater tumor growth (Fig. 9B-C). Similarly, the sizes of the tumors excised from the euthanized mice showed that the MSI2-silenced MDA-MB-231-injected group had the highest tumor load among all the groups, whereas the MSI2-knockdown clone #1 tumor-bearing group had the lowest tumor load (Fig. 9C-D). Additionally, the mRNA and protein levels of Ki67 and PCNA were significantly increased in MSI2-depleted MDA-MB-231 cells (Fig. S6A-D). These findings suggest that MSI2 plays different roles in maintaining the growth of MDA-MB-231 cells and that MSI2 downregulation may lead to uncontrolled cellular proliferation but not necessarily dysregulated cell migration and invasion.

*In vivo* imaging with an IVIS was performed to monitor cancer metastasis and revealed lung metastasis in mice implanted with clone #1; however, the other groups did not show any metastasis. MSI2 knockdown inhibited the metastasis of the injected clone #1 (Fig. S7). Interestingly, the tumor load (reflected by the luminescence intensity) was highest in the MSI2-silenced MDA-MB-231 tumor-implanted group, which was consistent with previous observations (Fig. S7). On the basis of the *in vivo* imaging results, mouse lungs were processed for evaluation of metastasis. Enlarged mouse lungs with uneven morphology (metastatic nodules) were clearly observed in the Scr-treated clone #1 tumor-bearing group, whereas the number of nodules was reduced in the MSI2-knockdown group (Fig. 9E). IHC staining of mouse lungs revealed upregulation of MSI2, vimentin, and N-cadherin and downregulation of E-cadherin in the Scr-treated clone #1 tumor-bearing group, whereas these changes were reversed when MSI2 was silenced. Additionally, the presence of metastasized TNBC cells in mice lungs was confirmed through detection of human breast cancer cell specific protein human leukocyte antigen (HLA) class I HLA-ABC expression validating positive metastasis of control and DEHP-exposed cells to mice lungs (Fig 9F).

Histological evaluation of the metastatic nodules was conducted via HE and IHC staining. Veterinary pathology revealed lymph node (LN) metastasis in 67% of the mice in the Scr-treated MDA-MB-231 and DEHP-exposed clone #1 tumor-bearing groups. Notably, the rate of LN metastasis decreased to 50% in the MSI2-silenced clone #1 tumor-bearing group. Furthermore, 100% of the mice in the MDA-MB-231 (Scr-treated, MSI2-depleted) and Scr-treated clone #1 tumor-bearing groups exhibited tumor growth in the mammary fat pad; however, only 50% of the mice in the MSI2-depleted clone #1 tumor-bearing group exhibited discernable tumor growth (Table S2). Histological observations revealed disruption of the karyoplasmic ratio (cell-to-nucleus size ratio) in both the lymph nodes and the MFP of Scr-treated clone #1 tumor-bearing mice. Enlarged nuclei indicative of metastasis in breast cancer were observed in Scr-treated clone #1 tumor-bearing mice; however, MSI2 knockdown restored the karyoplasmic ratio to normal (Fig. S8A-B). Overall, MSI2 knockdown was shown to reduce metastasis in mice bearing tumors derived from DEHP-exposed clone #1. Interestingly, MSI2 knockdown in control MDA-MB-231 cells increased cell proliferation, indicating that MSI2 expression in control MDA-MB-231 cells may be required for normal cell growth; however, in cells subjected to prolonged DEHP exposure, MSI2 overexpression decreased proliferation but increased metastatic potential.

### Increased MSI2 expression is indicative of poor prognosis in breast cancer patients

Analysis of a TCGA dataset was employed to evaluate the clinical implications of MSI2 expression in BC patients. The MSI2 expression levels in 903 tumor tissues and 103 adjacent normal tissues were significantly increased (Table 5, Fig. S9). Furthermore, we evaluated the relationships between MSI2 expression and both OS and relapse-free survival (RFS) in BC patients from the TCGA dataset. High MSI2 expression was associated with poor OS with lymph node metastasis [crude hazard ratio (CHR) =1.44, adjusted hazard ratio (AHR) = 1.45, 95% confidence interval (CI) = 0.88-2.39] and poor RFS with lymph node metastasis (CHR =1.50, AHR = 1.58, 95% CI = 0.62-4.02) (Table S3). These results indicate that high MSI2 levels are indicative of poor prognosis in BC patients.

## Discussion

The widespread daily use of plastic products increases the risk of disease due to phthalate exposure. Despite prevalent human exposure to phthalates, the metabolism and excretion of phthalates from the human body are rapid, and these compounds may not present any serious threat. However, phthalate exposure beyond tolerable daily intake (TDI) and the duration of exposure may surpass the metabolic and excretion rates 42. The detrimental effects of phthalates are well studied; nevertheless, most studies have focused on short-term exposure to high concentrations of phthalates. To better understand the impact of physiological phthalate exposure, long-term studies are necessary. In an attempt to replicate physiological exposure conditions, we designed experiments focused on long-term DEHP exposure at the physiological level in BC cells.

Among phthalates, DEHP plays crucial roles in regulating cancer growth, proliferation, angiogenesis, and metastasis. DEHP reportedly regulates cell migration and invasion in several solid cancers. MEHP, a metabolite of DEHP, was found to activate MMP2 expression to induce the invasion and migration of human testicular embryonal carcinoma cells 43. Similarly, DEHP treatment at low concentrations induced cell motility and invasion of the human neuroblastoma cell line 44. Our results revealed increased migration and invasion of MDA-MB-231 cells following DEHP treatment; however, decreased migration and invasion were observed in MCF7 cells. Several studies have described DEHP as a regulator of cell proliferation. For example, DEHP was shown to mediate an increase in the proliferation of HepB2 liver cancer cells through the activation of PI3k/Akt signaling 45. Unexpectedly, prolonged DEHP treatment attenuated the proliferation of MDA-MB-231 cells. We also performed an *in vivo* zebrafish xenograft assay to evaluate cell migration, which revealed increased metastatic potential of long-term DEHP-exposed clone #1 cells compared with that of untreated MDA-MB-231 cells. Overall, the effect of prolonged DEHP treatment at low concentrations differed from that of acute DEHP treatment at high concentrations. Furthermore, next-generation sequencing (NGS) analysis of untreated MDA-MB-231 cells and DEHP-exposed clones was performed to determine the differential gene expression and underlying mechanism involved. GO analysis of the DEGs revealed enrichment of biological functions such as locomotion, positive regulation of locomotion, regulation and cell migration and motility. The IPA of the NGS data also revealed that cell movement and development were the top cellular functions. The findings of the NGS analysis were consistent with those of previous reports.

MSI2, an RNA-binding protein, has been widely studied in different cancer types, and its regulation is crucial for disease development. The presence of a poly-A-protein binding site and two RNA recognition motifs enables MSI2 to act as a posttranscriptional regulator of several genes that determine cell fate 46. According to previous reports, MSI2 can interact with mRNAs and regulate the translation of several oncogenic proteins related to cancer signaling pathways, such as the TGFβ/SMAD, mTOR, PI3K/Akt, cMET, NUMB/Notch, cMyc, and Hh signaling pathways 47. Our IPA results of the NGS data predicted MSI2 activation; downstream analysis of cell migration indicated that MSI2 is a major regulator of DEHP-mediated migration of MDA-MB-231 cells. Various studies have documented a close association between MSI2 expression and EMT regulation. During EMT, epithelial cells acquire an elongated fibroblastic phenotype similar to that of mesenchymal cells 48. Similarly, we found that, compared with parental MDA-MB-231 cells, increased MSI2 expression in DEHP-exposed clones resulted in the acquisition of a mesenchymal-like elongated morphology. Compared with those in untreated MDA-MB-231 cells, the expression of the EMT markers α-SMA, β-catenin, SNAI1, and vimentin in DEHP-exposed clones was also increased.

Cancer migration and invasion are complex multistep processes that involve several small molecules for regulation. To facilitate the migration of tumor cells, changes in cellular adhesion and ECM rearrangement are necessary. MMPs) with proteolytic activity are major regulators of cellular migration. MMP9 contributes to the degradation of the ECM and basement membrane, acting as a facilitator of nodal invasion and metastasis 49. Several studies have shown that MMP9 is a mediator of cancer metastasis. In basal-like TNBC cells, MMP9 production and secretion regulate the malignant progression and metastasis of these cells 50. Another study revealed that L-theanine treatment reverses MMP9-mediated metastasis in prostate cancer by downregulating Snail and MMP9 expression 51. Interestingly, the expression and regulation of MMP9 are controlled by transcription factors, and MMP9 mRNA contains binding sites for several transcription regulators, such as NFκB, AP-l, Spl, and Ets-l 52. NFκB is closely associated with the regulation of MMP9 transcription and can be regulated by several intracellular signaling molecules. Curcumol was found to limit the MMP9-mediated invasion and migration of MDA-MB-231 cells by suppressing JNK1/2- and Akt-dependent NFκB/MMP9 signaling 53. Similarly, CoQ0 was found to reduce EMT by limiting PI3K/Akt/NFκB/MMP9 signaling in TNBC cells 54. Our results predicted the activation of TNF and NFκB signaling on the basis of MSI2-RNA-IP NGS data. Furthermore, GSEA revealed a positive interaction of DEHP-exposed clone #1 with TNF signaling and Akt signaling, which is consistent with the results of MSI2 RNA-IP NGS analysis. However, no significant change in TNFα mRNA or protein levels was observed, indicating that TNFα signaling might not be involved. Furthermore, western blotting and gelatin zymography revealed upregulation of PI3K/Akt/Ikk/NFκB/MMP9 signaling in DEHP-exposed clones. The transcriptional activity of NFκB was evaluated through IF staining, and western blotting revealed increased NFκB p65 in the cytosolic and nuclear fractions of DEHP-exposed clone #1 cells, whereas MSI2 depletion in DEHP-exposed clone #1 cells reduced the cytoplasmic expression and nuclear translocation of NFκB p65. Similarly, MSI2 knockdown reduced MMP9 activity in DEHP-exposed clones. Consistent with previous findings, these results indicate that prolonged DEHP treatment activates the PI3K/Akt/NFκB/MMP9 signaling axis regulated by MSI2; this axis participates in DEHP-mediated metastasis of MDA-MB-231 cells.

As a type III intermediate filament protein, vimentin maintains cellular morphology and provides anchor points for cell organelles 55. It is a widely distributed filament and is involved in EMT, cell adhesion, differentiation, migration, and cell invasion 56. Vimentin is generally considered an EMT marker, as intermediate vimentin filaments maintain cell shape (which facilitates the transition to a mesenchymal phenotype, an important step in metastasis initiation). In addition, at the molecular level, vimentin expression is regulated by Twist1, and vimentin further acts as a scaffold for Slug, a transcription factor important for EMT regulation 57, 58. Accumulating evidence highlights the importance of vimentin in mediating EMT and the metastasis cascade. Berr *et al.* demonstrated that vimentin-null mice exhibit restricted lung metastasis and delayed tumor progression. The loss of vimentin resulted in reduced expression of EMT and metastasis markers 59. Slug-mediated vimentin expression was reported to regulate EMT and maintain migratory activity in breast cancer 60. The role of vimentin in structural stability and cellular movement has been well studied, indicating that the polarization and distribution of vimentin intermediate filaments maintain cell motility. Vimentin-derived filaments reportedly provide a template for the microtubule network to increase cell polarity and cell migration. Vimentin depletion results in the loss of cellular polarity, affecting cell migration 61. Single-cell analysis of polyploid giant cancer cells revealed the uniform distribution of vimentin intermediate filaments across the cytoplasm, and compared with cells with tightly distributed vimentin filaments in the perinuclear space and nonpolarized vimentin filaments, those with polarization toward the leading edge of cells presented enhanced cell migration. In line with these findings, our results revealed that increased vimentin expression is regulated by MSI2 62. Moreover, the MSI2-vimentin interaction might be responsible for maintaining EMT potential and cell migration in DEHP-exposed clones. The IF results revealed a uniform distribution in the cytoplasm and polarization of vimentin intermediate filaments in DEHP-exposed MDA-MB-231 cells. Interestingly, MSI2 knockdown decreased vimentin expression and increased the perinuclear localization of vimentin in DEHP-exposed MDA-MB-231 cells. Overall, MSI2 interacts with and regulates vimentin expression, impacting its cellular distribution and polarization and controlling EMT potential and cell migration.

miRNAs are short noncoding RNAs that can regulate gene expression either by controlling translation or directly driving the degradation of target mRNAs. miRNA targeting is enabled by a 6-7 nucleotide seed sequence that can bind with a complementary sequence in the 3′UTR of the target mRNA, and the mode of activity depends on the complementarity of the miRNA seed sequence and the mRNA sequence 63. miR-155-5p is reported to play a role in survival, proliferation, chemoresistance, stem cell formation, migration and invasion in different cancers as either an oncomiR or a tumor suppressor 64-69. In breast cancer, miR-155-5p was found to suppress the malignant transformation of breast epithelial cells by targeting ErbB2 expression 70. Another study revealed that miR-155-5p inhibits the migration and invasion of prostate cancer cells through targeting SPCOK1, indicating the tumor-suppressive role of miR-155-5p 71. Our study revealed similar tumor suppressor activity of miR-155-5p in long-term DEHP-exposed MDA-MB-231 cells. The prediction of miRNAs that target MSI2 mRNA was performed via an online database, which revealed that miR-155-5p has a strong affinity for MSI2. Two conserved sites were identified in the 3′UTR of MSI2 mRNA. qPCR revealed significantly lower levels of miR-155-5p in DEHP-exposed clone #1 cells than in untreated cells. miR-155-5p mimic treatment downregulated MSI2 and vimentin in a dose-dependent manner, indicating that miR-155-5p specifically targeted MSI2 mRNA. Wound healing assays revealed that miR-155-5p mimic treatment inhibited MSI2-mediated cell migration in DEHP-exposed clone #1 cells. Furthermore, the results of the MSI2 3′UTR luciferase assay revealed that cells exhibited significantly lower luciferase activity after miR-155-5p treatment. Overall, prolonged DEHP treatment reduced the miR-155-5p level in MDA-MB-231 cells; conversely, miR-155-5p overexpression in untreated MDA-MB-231 cells might reduce the expression of MSI2 via either translational inhibition or mRNA degradation, eventually contributing to inhibited migration and invasion.

## Conclusion

In summary, our study revealed that long-term DEHP exposure at physiological concentrations facilitates the stemness and metastasis of TNBC cells. Mechanistically, DEHP exposure upregulated MSI2, which not only facilitates EMT by physically interacting with vimentin but also initiates the PI3K/Akt/NFκB/MMP9 axis to enhance metastasis. However, the effects of long-term DEHP exposure on TNBC progression are significantly abolished by genetic knockdown of MSI2. Notably, a reduced level of miR-155-5p is found in DEHP-exposed TNBC cells, and it negatively regulates MSI2 expression. Collectively, these results highlight that MSI2 might be a potential therapeutic target for TNBC treatment and could serve as a prognostic biomarker for patients with metastatic disease.

## Supplementary Material

Supplementary figures and tables.

## Figures and Tables

**Figure 10:**
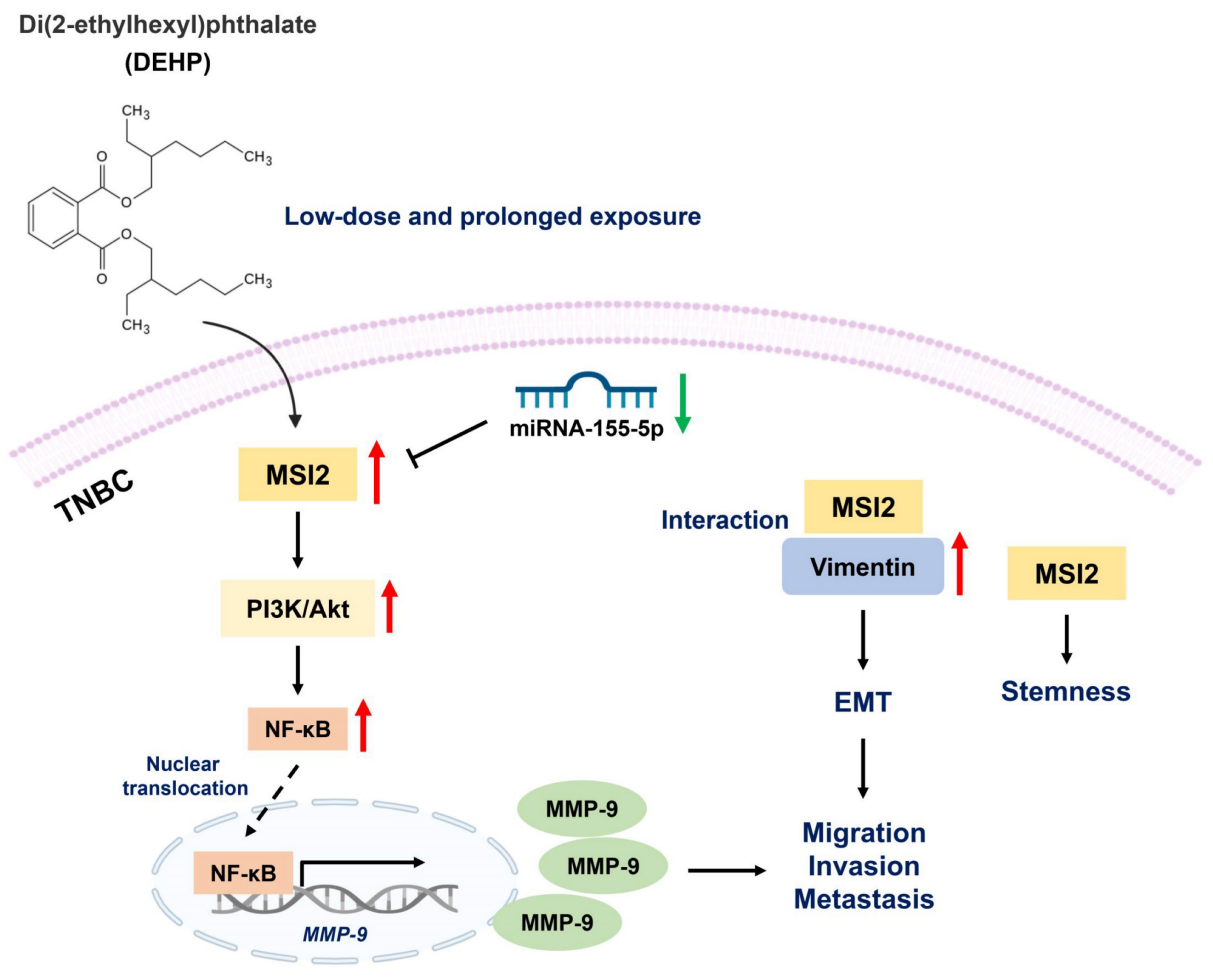
Schematic representation of DEHP-induced TNBC progression. Low-dose and prolonged DEHP exposure results in MSI2 overexpression in MDA-MB-231 cells. Increased MSI2 expression initiates the PI3K/Akt/NF-κB/MMP-9 axis and regulates vimentin to enhance EMT, which facilitates migration, invasion, and metastasis. In addition, MSI2 promotes TNBC stemness. Interestingly, miR-155-5p negatively regulates the expression of MSI2, while miRNA-155-5p is downregulated under DEHP treatment.

**Table 1:** Clone ID and target sequences of the scrambled and MSI2-targeting shRNAs

Name	Clone ID	Target Sequence
Scramble (Scr)	ASN0000000004	CTAAGGTTAAGTCGCCCTCG
MSI2 (shMSI2)	TRCN0000324685	CCCAACTTTGTGGCAACCTAT

**Table 2:** Quantitative PCR (qPCR) primer sequences

miR-155-5p	forward	5′- TTTGCCTCCAACTGACTCCT-3′
	reverse	5**′**- GCAGCAATTTGTTCCATGTG-3**′**
U6-snRNA	forward	5**′**- CTCGCTTCGGCAGCACA-3**′**
	reverse	5**′**- AACGCTTCACGAATTTGCGT-3**′**

**Table 3:** Quantitative PCR (qPCR) primer sequences

MSI2	forward	5′-ATCCCACTACGAAACGCTCC-3′
	reverse	5′-GGGGTCAATCGTCTTGGAATC-3′
Vimentin	forward	5′-TGCCGTTGAAGCTGCTAACTA-3′
	reverse	5′-CCAGAGGGAGTGAATCCAGATTA-3′
β-actin	forward	5′-TGAGACCTTCAACACCCCAGCCAT-3′
	reverse	5′-CGTAGATGGGCACAGTGTGGGTG-3′

**Table 4:** qPCR primer sequences for proliferation markers

Ki67	forward	5′-TTCGCAAGCGCATAACCCA-3′
	reverse	5′-AACCGTGTCACAGTGCCAAA-3′
PCNA	forward	5′-ACACTAAGGGCCGAAGATAACG-3′
	reverse	5′-ACAGCATCTCCAATATGGCTGA -3′
β-actin	forward	5′-TGAGACCTTCAACACCCCAGCCAT-3′
	reverse	5′-CGTAGATGGGCACAGTGTGGGTG-3′

**Table 5:** Comparisons of MSI2 expression between tumor and adjacent normal tissues from breast cancer patients

Variables	Adjacent normal tissue		Tumor tissue		*P* value*
	Mean ± SD (n=103)	Median	Mean ± SD (n=903)	Median	
MSI2 expression	7.69 ± 0.84	7.9000	8.66 ± 0.98	8.7246	< 0.001

Abbreviations: SD, standard deviation. *p values were estimated via Student's t test.

## Abbreviations

AHR: adjusted hazard ratio
AIG: anchorage-independent growth
AIG: anchorage-independent growth
cDNA: complementary DNA
CHR: crude hazard ratio
CI: confidence interval
CoIP: coimmunoprecipitation
Ctrl: control
DEG: differentially expressed gene
Dox: doxorubicin
DoxR: doxorubicin resistant
DEHP: Di(2-ethylhexyl) phthalate
EMT: epithelial-mesenchymal transition
GO: gene ontology
GSEA: gene set enrichment analysis
HE: hematoxylin and eosin
hpf: hours postfertilization
hpi: hours postinjection
IACUC: institutional animal care and use committee
IF: immunofluorescence
IHC: immunohistochemistry
IPA: ingenuity pathway analysis
LC/MS/MS: liquid chromatography/mass spectrometry
LN: lymph nodes
MFP: mammary fat pad
mRNA: messenger RNA
NGS: next-generation sequencing
OS: overall survival
RFS: relapse-free survival
RNA-IP: RNA immunoprecipitation
PBS: phosphate-buffered saline
ROC: receiver operating characteristic
Scr: scramble
SD: standard deviation
SIV: subintestinal vessel
TCGA: Cancer Genome Atlas
miRNA: microRNA
MSI2: musashi RNA-binding protein 2
qPCR: quantitative real-time polymerase
