# Supplementary material for: Prolonged DEHP exposure enhances the stemness and metastatic potential of TNBC cells in an MSI2-dependent manner
Source: Int J Biol Sci. 2025 Feb 3;21(4):1705–29. doi: 10.7150/ijbs.101598 (PMC11844279; doi:10.7150/ijbs.101598)
Supplement: Supplementary file 1 — Supplementary figures and tables. [file ijbsv21p1705s1.pdf]

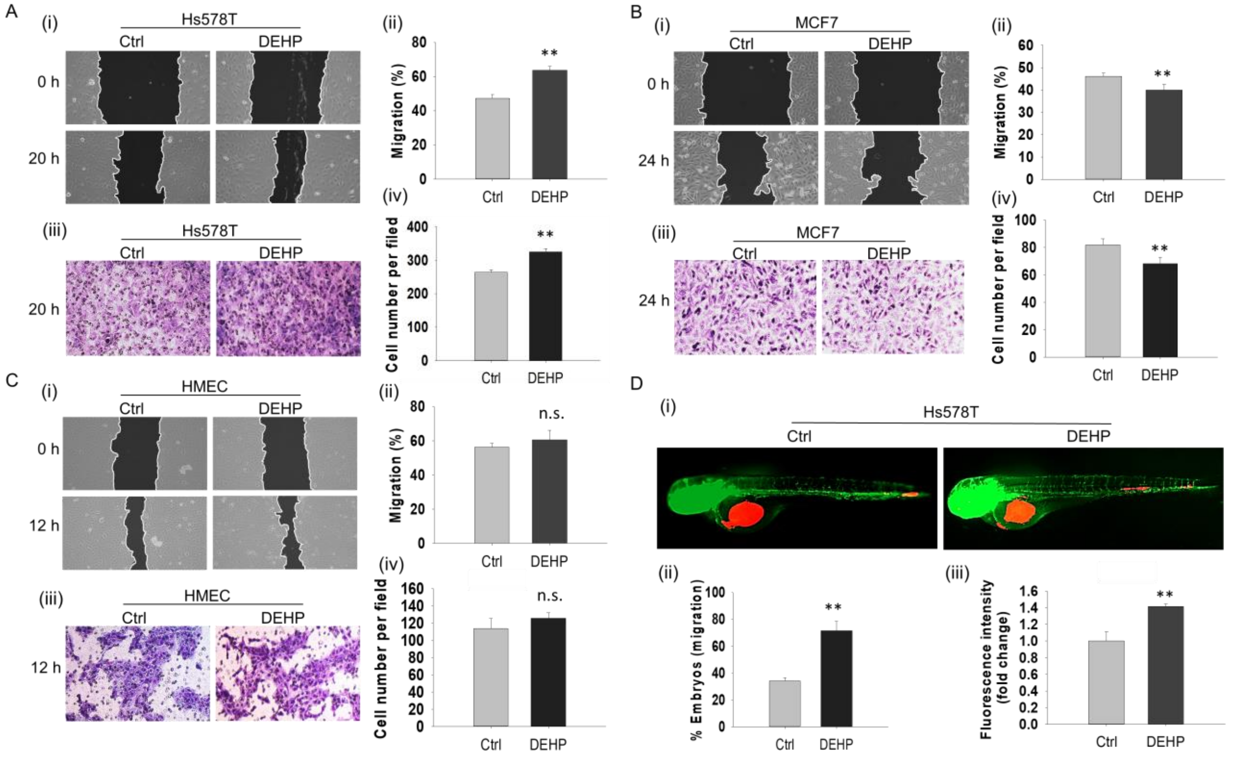

Figure S1. Long term DEHP exposure induce migration and invasion in triple negative Hs578T cells but not in tumorogenic MCF7 and normal mammary epithelial HMEC cells. **(A-i)** Cell migration of control and DEHP-exposed Hs578T cells was evaluated by wound healing assay for 20 h; **(A-ii)** Quantitative analysis of cell migration (means ± SD); **(A-iii)** Cell invasion was accessed by matrigel coated transwell inserts for 20 h in control and DEHP exposed Hs578T cells; **(A-iv)** Quantitative analysis of cell invasion (means ± SD). **(B-i)** Cell migration of control and DEHP exposed MCF7 cells was evaluated by wound healing assay for 24 h; **(B-ii)** Quantitative analysis of cell migration (means ± SD); **(B-iii)** Cell invasion was accessed by matrigel coated transwell inserts for 24 h in control and DEHP exposed MCF7 cells; **(B-iv)** Quantitative analysis of cell invasion (means ± SD). **(C-i)** Cell migration of control and DEHP exposed HMEC cells was evaluated by wound healing assay for 12 h; **(C-ii)** Quantitative analysis of cell migration (means ± SD); **(C-iii)** Cell invasion was accessed by matrigel coated transwell inserts for 12 h in control and DEHP exposed HMEC cells; **(C-iv)** Quantitative analysis of cell invasion (means ± SD). **(D-i)** Zebrafish xenograft assay to confirm *in vivo* cell migration of Hs578T cells in Tg (*fli1: EGFP*) zebrafish embryos (fluorescence imaging obtained at 24 hpi). **(D-ii)** Quantification of the percentage of zebrafish embryos showing cell migration to SIV (means ± SD, n=50); **(D-iii)** Quantitative analysis of cell migration to the zebrafish SIV (fluorescence intensity indicative of cell number, fold change vs control); ** $P < 0.001$; n.s.: non-significant.

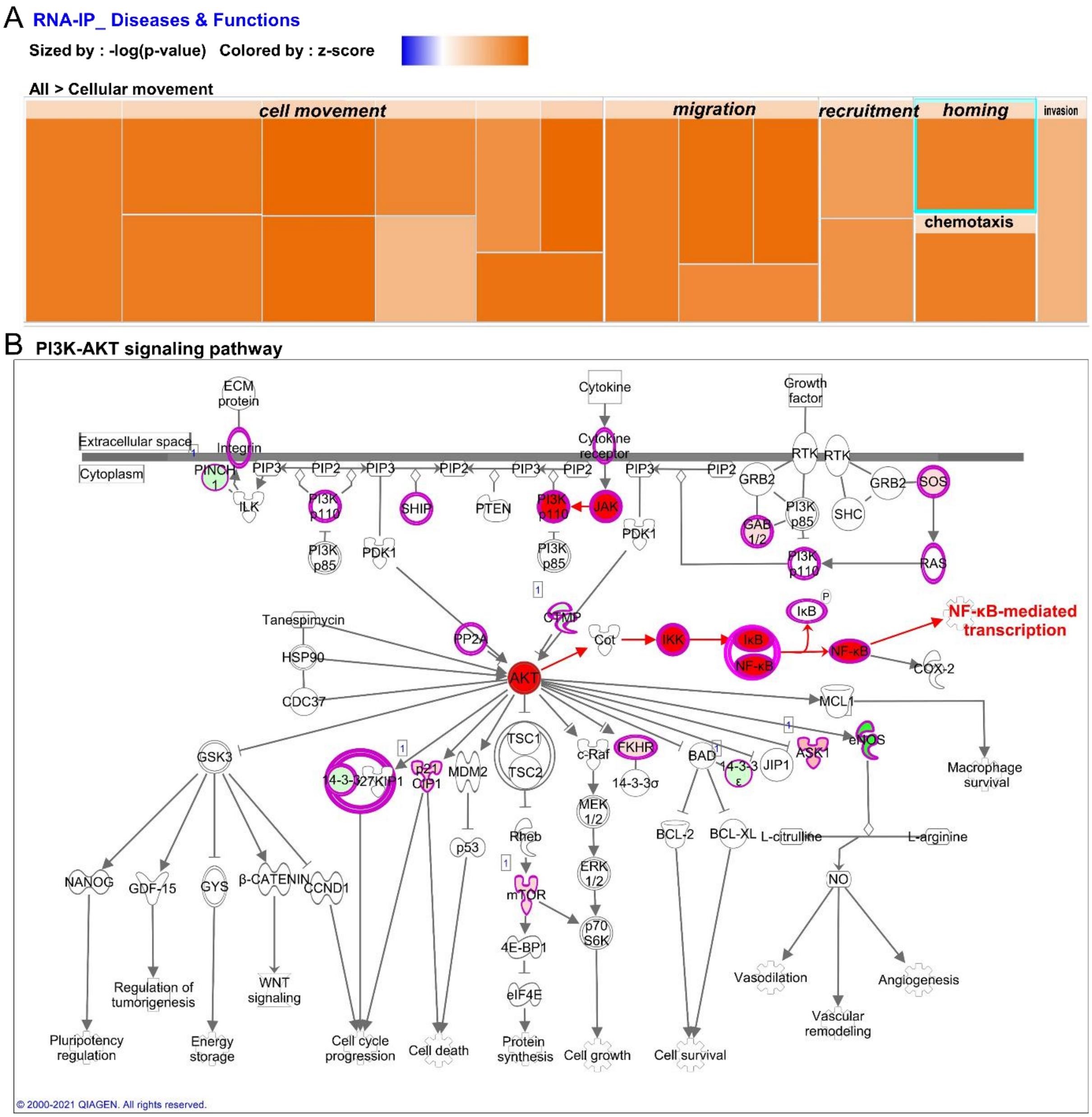

Figure S2. Involvement of MSI2 mediated PI3K/Akt/NFκB signaling axis in DEHP induced metastasis predicted by NGS analysis. **(A)** Disease and Function (cell movement) analysis of RNA-IP NGS data performed by IPA. Chart distribution, sized by: -log(p-value); colored by: z-score. **(B)** Enrichment of PI3K/Akt signaling pathway evaluated by pathway prediction in IPA. (red: upregulation; green: downregulation; red arrows: activated signaling, violet: observed expression change).

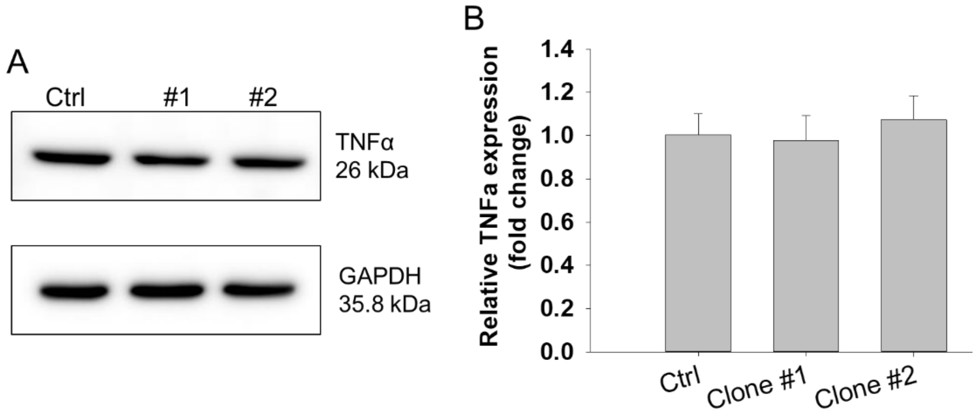

Figure S3. Long term DEHP exposure have no significant effect on TNFα expression. (A) Evaluation of TNFα expression in MDA-MB-231, DEHP exposed clone #1 and #2 performed by western blotting. **(B)** Evaluation of TNFα mRNA levels in MDA-MB-231, DEHP exposed clone #1 and #2 (means ± SD).

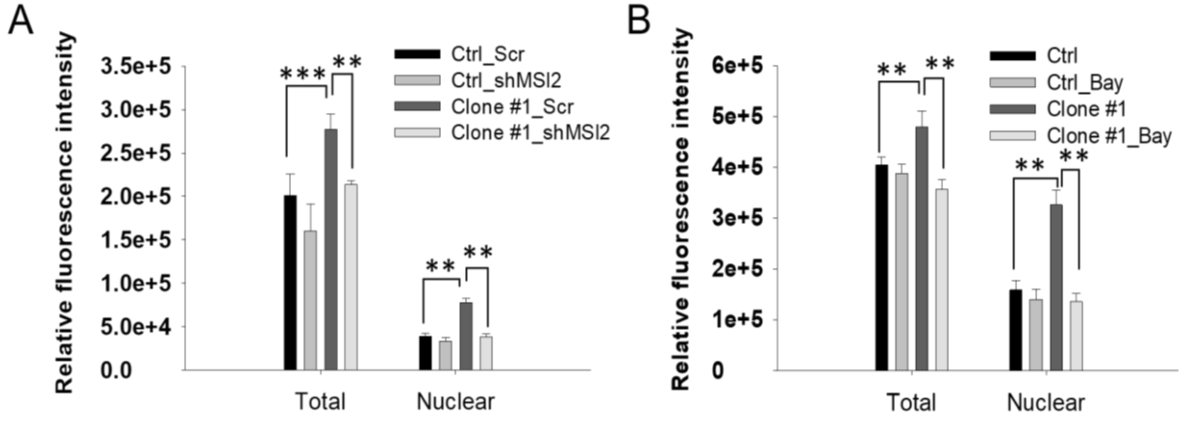

Figure S4. MSI2 depletion, BAY11 7082 reduce total and nuclear NFκB expression in DEHP exposed clone #1. (A) Quantitative analysis of NFκB expression (fluorescence intensity) in scr-treated and MSI2 depleted MDA-MB-231 and DEHP exposed clone #1 (means ± SD). **(B)** Quantitative analysis of NFκB expression (fluorescence intensity) in control and BAY 11 7082 treated MDA-MB-231 and DEHP exposed clone #1 (means ± SD). **$P < 0.001$.

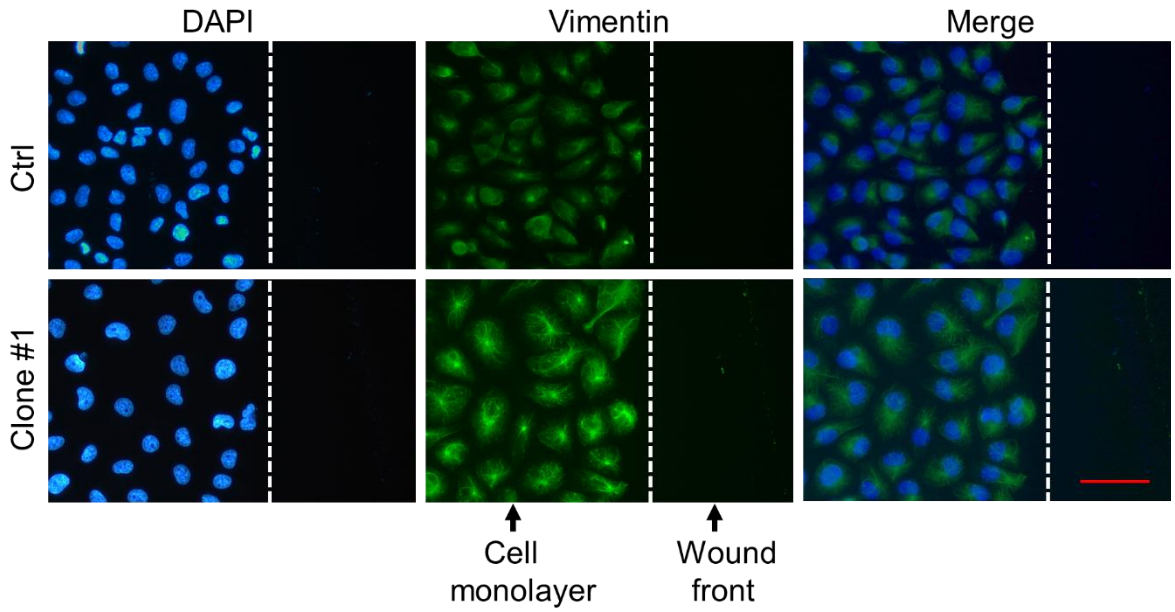

Figure S5. Polarization of vimentin filaments drives cell migration in DEHP exposed clone #1. Evaluation of vimentin filament polarization by IF in wound healing assay after 12 h of wound formation in MDA-MB-231 and DEHP exposed clone #1. Scale bar = 70 µm.

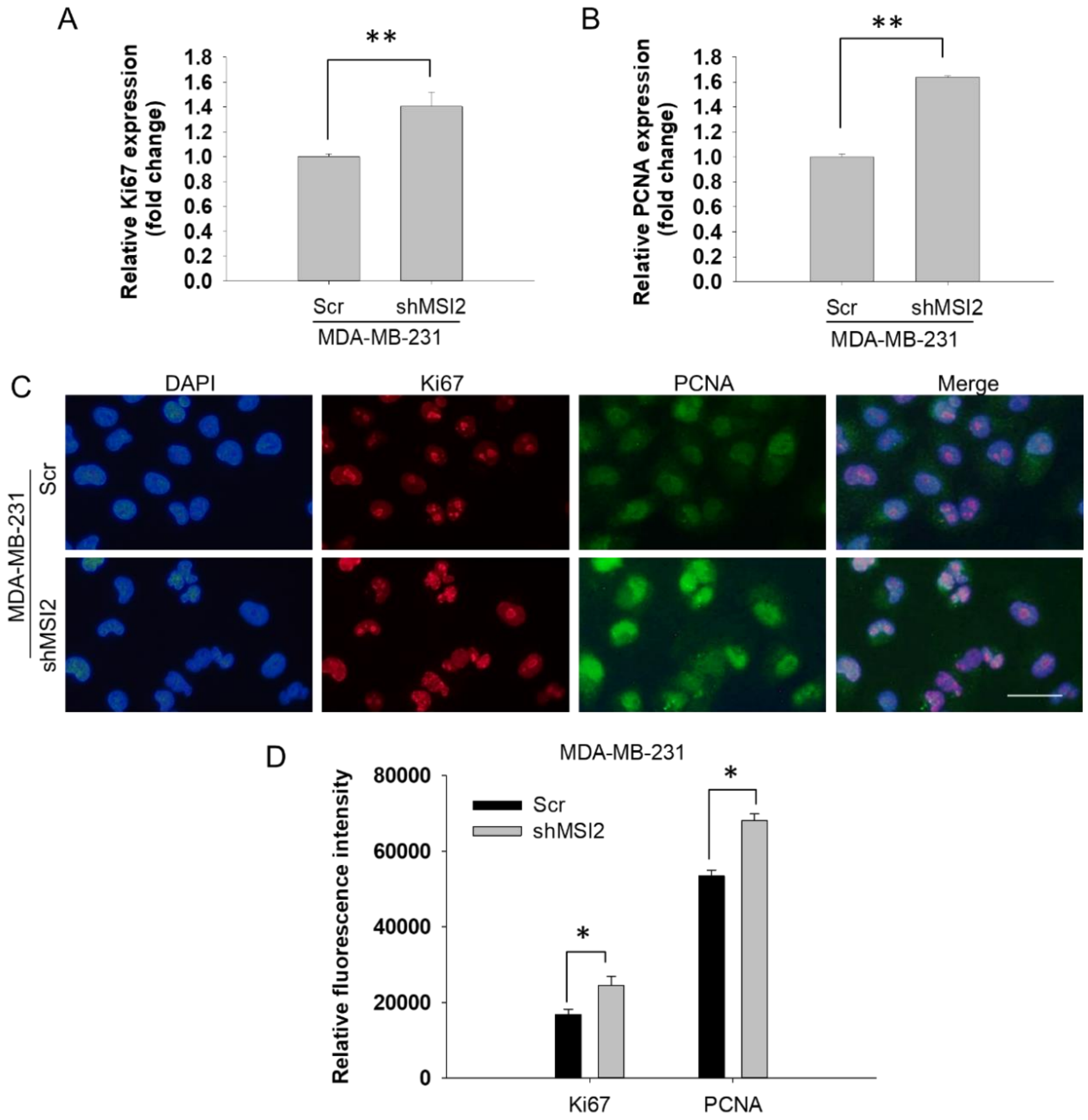

Figure S6. MSI2 depletion induce proliferation marker expression in MDA-MB-231 cells. (A) Evaluation of proliferation markers Ki67 mRNA levels in Scr-treated and MSI2 depleted MDA-MB-231 (means ± SD). **(B)** Evaluation of proliferation markers PCNA mRNA levels in Scr-treated and MSI2 depleted MDA-MB-231 (means ± SD). **(C)** Immunofluorescence (IF) analysis of intracellular expression of Ki67 (red) and PCNA (green). Nuclear staining (blue). Scale bar = 50 µm. **(D)** Quantitative analysis of Ki67 and PCNA expression (fluorescence intensity) in Scr-treated and MSI2 depleted MDA-MB-231 cells (means ± SD); *P<0.05, **P < 0.001.

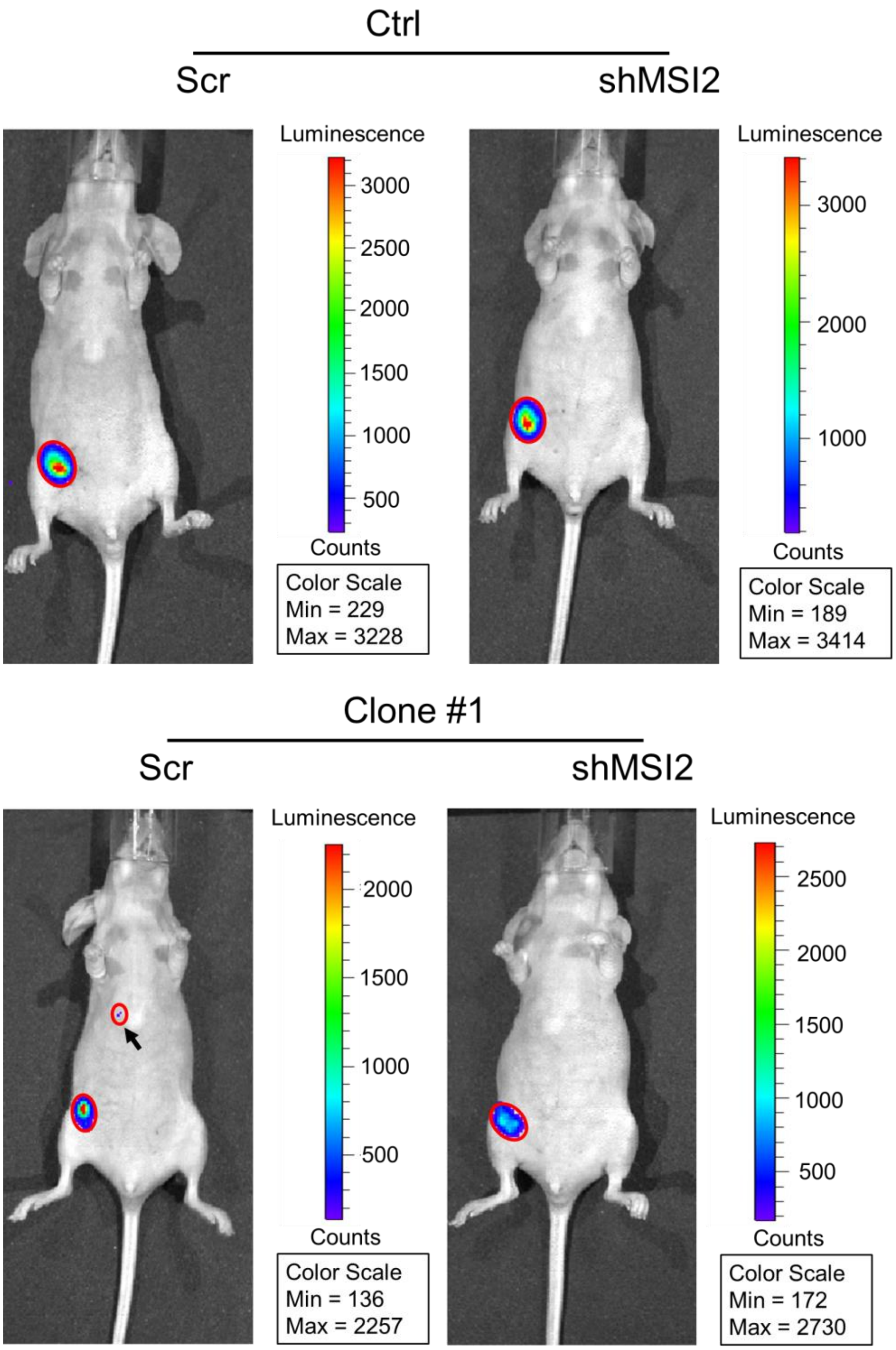

Figure S7. *In vivo* breast cancer metastasis tracking performed by high-content IVIS spectrum imaging in MDA-MB-231 and DEHP-exposed clone #1 (Scr-treated and MSI2-depleted) tumor-implanted mice.

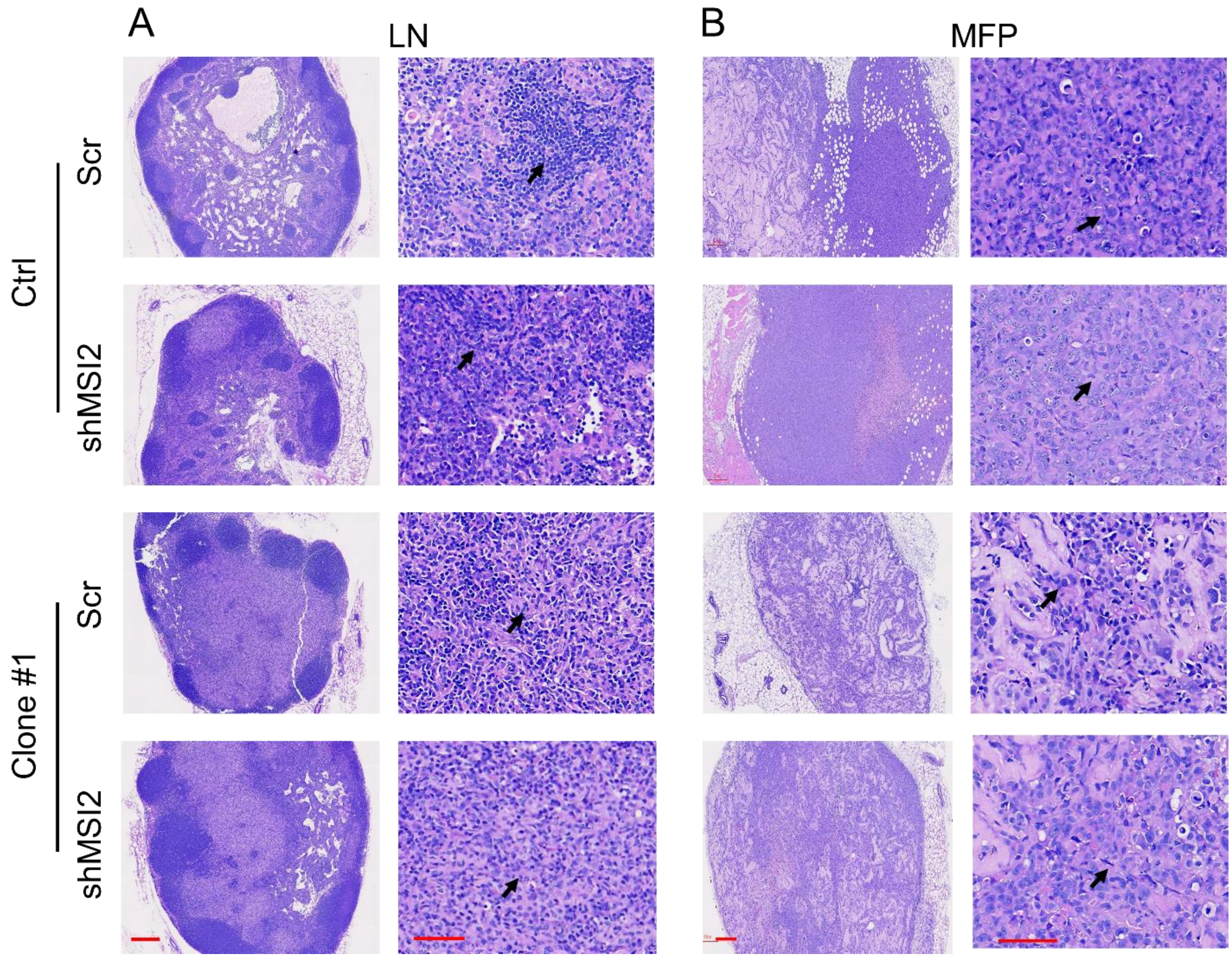

Figure S8. MSI2 knockdown reduce DEHP induced breast cancer lung metastasis. (A) Evaluation of lymph node (LN) metastasis by HE staining of excised lymph nodes. **(B)** Mammary fat pad (MFP) metastasis evaluation performed by HE staining of excised mammary fat pad**.** Scale bar = 300 µm (zoom out); Scale bar = 60 µm (zoom in).

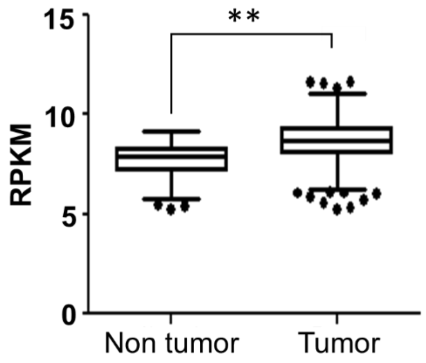

Figure S9. High MSI2 expression in tumor tissues of breast cancer patients. Increased expression of MSI2 in tumor tissue of breast cancer patients evaluated by TCGA based RNA seq. [means ± SD, n=103 (non-tumor); n=903 (tumor)]; ** P < 0.001.

Table S1. Vimentin identified as shared protein in MSI2 flag CoIP. Peptide sequencing and protein identification performed by LC/MS/MS analysis based on score, coverage and relative abundance among identified proteins.

Sample	Accession number	Description	Score	Coverage	Unique Peptides	#PSMs	Relative abundance (%)
Ctrl	XP_006717563.1	vimentin isoform X1 [Homo sapiens]	1088.30	69.10	28	54	2.1
	Peptide sequence	*Protein accession number*	*# PSMs*	*q-value*	*Ion score*	*m/z ratio [Da]*	*RT [min]*
	FANYIDK	XP_006717563.1	1	0.005	11	435.7213	22.33
Clone #1	XP_006717563.1	vimentin isoform X1 [Homo sapiens]	5762.42	89.48	63	294	14.7
	Peptide sequence	*Protein accession number*	*# PSMs*	*q-value*	*Ion score*	*m/z ratio [Da]*	*RT [min]*
	FANYIDK	XP_006717563.1	2	0.004	17	435.7211	22.11

Abbreviations: Score, Protein score (MudPIT score), sum of the scores of peptides; Coverage, Sequence coverage of identified protein (%); #PSMs, Total number of peptide-spectra matches; Relative abundance, Estimated protein abundance by spectrum counting;

Peptide sequence, Amino acid sequence of identified peptide; #PSMS, Total number of spectrums matches for the identified peptide; Ion score, Mascot ion score for identified peptide; m/z ratio, Ratio of peptide mass and peptide charge in MS, RT, The retention time of the identified peptide in LC-MS analysis

Table S2. Histopathologic findings of HE staining of mammary fat pad and lymph nodes.

Group		Histopathologic findings	
		MFP	LN
Ctrl (N=6)	Scr	6/6	4/6
	shMSI2	6/6	5/6
Clone #1 (N=6)	Scr	6/6	4/6
	shMSI2	3/6	3/6

Abbreviations: ***N****, number of mice in each group; MFP, mammary fat pad; LN, lymph node*

Scr, scrambled/mock shRNA treated; shMSI2, MSI2 knockdown

Table S3. Impact of MSI2 expression levels on survival by the clinicopathologic factors in breast cancer patients.

Variable	ROC	No. (%)	CHR (95% CI)	*p value**	AHR (95% CI)	*p value*†
**Overall survival**						
N0	Low	355 (81.4)	1.00		1.00	
	High	81 (18.6)	1.61 (0.77-3.39)	0.208	1.59 (0.76-3.35)	0.221[c]
N1, N2, N3	Low	369 (81.5)	1.00		1.00	
	High	84 (18.5)	1.44 (0.87-2.38)	0.154	1.45 (0.88-2.39)	0.151[c]
**Relapse-free survival**						
N0	Low	341 (91.9)	1.00		1.00	
	High	30 (8.1)	1.41 (0.42-4.73)	0.579	1.44 (0.43-4.85)	0.553[c]
N1, N2, N3	Low	330 (92.2)	1.00		1.00	
	High	28 (7.8)	1.50 (0.59-3.78)	0.394	1.58 (0.62-4.02)	0.333[c]

Abbreviations: ROC, receiver operating characteristics; CHR, crude hazard ratio; CI, confidence interval; AHR, adjusted hazard ratio.

**p values were estimated by Cox's regression; †p values were estimated by multivariate Cox's regression; [a]djusted for T classification (T3, T4 vs T1+T2); [b]adjusted for N classification (N1, N2, N3 vs N0); [c]adjusted for T classification (T3, T4 vs T1+T2)*
